# Measurement of suction pressure dynamics of sea lampreys, *Petromyzon marinus*

**DOI:** 10.1371/journal.pone.0247884

**Published:** 2021-04-27

**Authors:** Hongyang Shi, Christopher M. Holbrook, Yunqi Cao, Nelson Sepúlveda, Xiaobo Tan

**Affiliations:** 1 Department of Electrical and Computer Engineering, Michigan State University, East Lansing, Michigan, United States of America; 2 U. S. Geological Survey, Great Lakes Science Center, Hammond Bay Biological Station, Millersburg, Michigan, United States of America; 3 College of Control Science and Engineering, Zhejiang University, Hangzhou, Zhejiang, China; Beihang University, CHINA

## Abstract

Species-specific monitoring activities represent fundamental tools for natural resource management and conservation but require techniques that target species-specific traits or markers. Sea lamprey, a destructive invasive species in the Laurentian Great Lakes and conservation target in North America and Europe, is among very few fishes that possess and use oral suction, yet suction has not been exploited for sea lamprey control or conservation. Knowledge of specific characteristics of sea lamprey suction (e.g., amplitude, duration, and pattern of suction events; hereafter ‘suction dynamics’) may be useful to develop devices that detect, record, and respond to the presence of sea lamprey at a given place and time. Previous observations were limited to adult sea lampreys in static water. In this study, pressure sensing panels were constructed and used to measure oral suction pressures and describe suction dynamics of juvenile and adult sea lampreys at multiple locations within the mouth and in static and flowing water. Suction dynamics were largely consistent with previous descriptions, but more variation was observed. For adult sea lampreys, suction pressures ranged from –0.6 kPa to –26 kPa with 20 s to 200 s between pumps at rest, and increased to –8 kPa to –70 kPa when lampreys were manually disengaged. An array of sensors indicated that suction pressure distribution was largely uniform across the mouths of both juvenile and adult lampreys; but some apparent variation was attributed to obstruction of sensing portal holes by teeth. Suction pressure did not differ between static and flowing water when water velocity was lower than 0.45 m/s. Such information may inform design of new systems to monitor behavior, distribution and abundance of lampreys.

## Introduction

Species-specific monitoring activities, such as animal detection [1], behavior observation [2], population assessment [3] and habitat use evaluation [4] represent fundamental tools for natural resource management and conservation. In aquatic environments, monitoring methods have long relied on sonar imaging to characterize distributions and densities of groups and capture methods to identify individuals [5, 6] Emerging technologies promise to overcome many of the inherit limitations of those methods. For example, autonomous underwater vehicles (AUV) can be used to collect multimedia data and detect animals through image processing [3]. However, automated species identification remains a great challenge and may not be practical in some environments [7]. Environmental DNA (eDNA) [8, 9] has also become popular for species-level monitoring, but may be limited to species-level occupancy and be subject to false positives caused by transport of tissues by currents and other processes in natural environments [10]. Therefore, more effective monitoring techniques that take advantage of species-specific characteristics are desired.

Sea lampreys (*Petromyzon marinus*) are anadromous fish native to the Atlantic Ocean, that invaded the Laurentian Great Lakes in the early 1900s, caused great harm to native fish communities [11–13], and triggered formation of a bi-national, basin-wide population control program. Success of the control program has been attributed to use of barriers, traps, sterilization, and lampricides [14, 15]. In Europe and North America, attempts to conserve and restore native lamprey populations have included dam removal [16] and artificial propagation [17]. In either case, strategies target individuals in life stages and habitats where they are most vulnerable to perturbations based on knowledge of species ecology and life history. Sea lampreys are basal vertebrates with a life history comprised of distinct larval, juvenile, and adult stages [18–20]. Larval sea lampreys burrow into stream sediment and feed on micro-organisms for 3~5 years. They then undergo a drastic metamorphosis into the juvenile stage with a powerful suctorial mouth, migrate downstream into the Atlantic Ocean or a Laurentian Great Lake, and parasitize fish for about 1.5 years [21], killing 18 kg of host fishes on average during that time [22]. Next, adult sea lampreys migrate upstream in the spring, where they will spawn and die. Although adult sea lampreys do not feed, they rely on oral suction during migration and spawning, for station-holding, nest construction, competition for mates, and mating [23].

Although many fishes use oral suction to feed, the ability to attach to surfaces using oral suction is unique to lampreys among freshwater fishes in the northern hemisphere (others include suckermouth catfishes of the genus *Hypostomus*, which are native to South America). When a lamprey approaches the desired surface, its annular muscle contracts and the buccal funnel spreads over the surface. The tooth-studded oral disc conforms to the surface and completes a seal, and then the armed tongue retracts into the oral passage and seals off the buccal funnel from the pharyngeal cavity. With the expansion of the buccal cavity, a partial vacuum inside this cavity and a corresponding suction force are created, which maintains a suction attachment. Next, water is pushed out of the pharyngeal cavity to the velar-sealed branchial cavity through the compression of an inner sinus, which increases vacuum pressure within the pharyngeal cavity. Once the tongue protracts, the vacuum pressure spreads into the buccal cavity and thereby forms a stronger suction [24]. Over time, suction pressure decreases due to leakage and pressure must be re-applied. Therefore, sea lamprey suction dynamics are characterized as intermittent cycles of rapid “pumps” (application of suction) separated by periods of leakage (loss of suction).

Although oral suction is a prominent characteristic of lampreys, it has not been exploited for lamprey management or conservation. Knowledge of specific characteristics of sea lamprey suction (e.g., amplitude, duration, and pattern of suction events; hereafter ‘suction dynamics’) may be useful to develop devices that detect, record, and respond to the presence of sea lamprey at a given place and time or as feeding devices for aquaculture. Apart from the suction mechanism, few researchers have measured the suction pressures created by sea lampreys. Gradwell [25] inserted cannulas into gill pouches and naris of four pre-spawning adult lampreys and measured the hydrostatic pressures of lampreys using a pressure transducer connected to each cannula. To obtain the pressure exerted by the spawning-run lampreys on an acrylic surface, Adams [24] connected an absolute pressure sensor to a port in the designated attachment area on the acrylic flange. However, the suction dynamics of adult lampreys and juvenile lampreys in flowing water is yet to be described. Additionally, it is not known if measurement of oral suction is sensitive to measurement location within the mouth (i.e., uniformity of vacuum pressure across the mouth). A pressure sensing panel which can measure the biological suction pressure, if deployed in tributaries or on dams, has the potential for sea lamprey detection, population assessment, and facilitation of lamprey passage or blockage. For instance, it could be deployed in a fishway to detect the sea lamprey according to the suction pressure change when it attaches, and to trigger action to block passage or repel the lamprey. It could also be deployed in streams to determine timing of stream entry and upstream migration, and to describe refuge habitat. Measurement of biological suction pressure dynamics, such as pressure amplitude, frequency, and suction duration, might also indicate condition, life stage, body size, or sex of sea lampreys if those dynamics are related to those biological characteristics. Moreover, the understanding of sea lampreys’ suction dynamics can improve design of next generation soft pressure sensors, which are described to be more biocompatible and convenient for deployment. Current pressure-sensing technologies are mainly categorized into five types: (1) piezoresistive [26, 27], (2) capacitive [28, 29], (3) nanogenerator-based [30, 31], (4) transistor-based [32, 33], and (5) fiber-optics [34, 35] pressure sensors, but these technologies have not been used to develop systems for the detection and monitoring of underwater sea lampreys.

We developed an effective pressure sensing panel, comprised of arrays of piezoresistive vacuum pressure sensors, to characterize suction dynamics of sea lampreys under water. Objectives of this study were: (1) to record and describe the range and distribution of suction pressures exerted by individual lampreys at two life stages (juvenile and adult); (2) to describe the frequency of suction events (re-pressurizing “pumps”); (3) to determine if vacuum pressure varies spatially across the sea lamprey mouth; and (4) to determine if suction dynamics (i.e., pressure ranges; event frequencies) differ when the sea lamprey is in static vs. flowing water.

## Methods

### The pressure-sensing apparatuses

**Fig 1:**
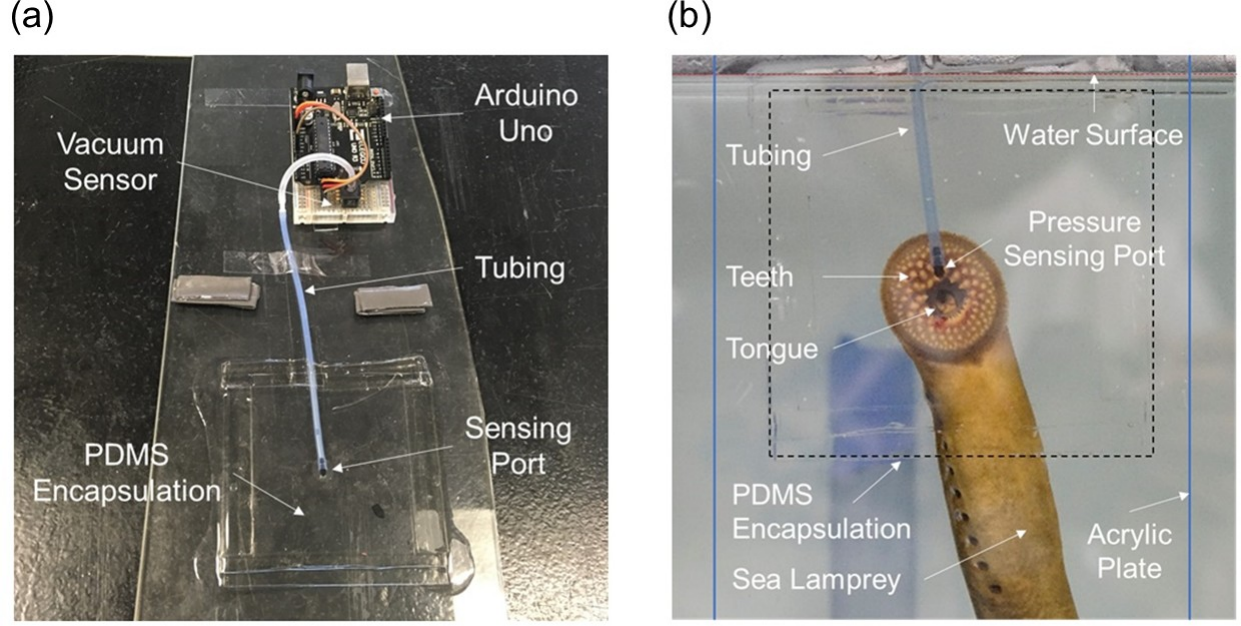
The single-port pressure sensing system used to measure oral suction pressures of sea lamprey, *Petromyzon marinus*. (a) The single-port sensing panel with a port on the acrylic plate encapsulated with polydimethylsiloxane (PDMS) and connected to the vacuum sensor beside the Arduino Uno microcontroller board via soft tubing, and (b) an adult sea lamprey attached to the sensing panel at the sensing port in a water-filled tank.

**Fig 2:**
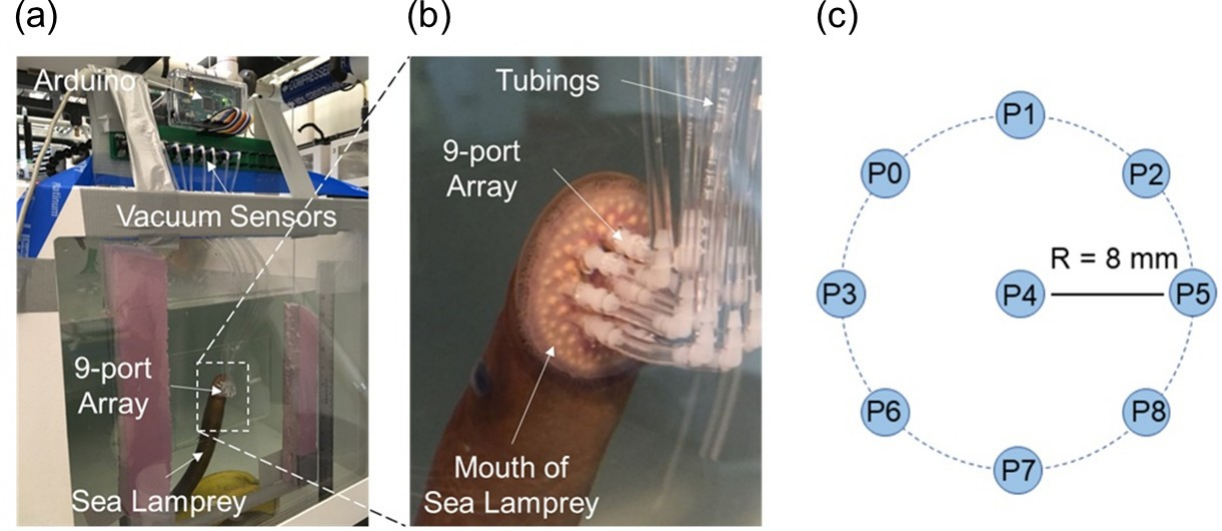
The 9-port circular pressure sensing system used to measure oral suction pressures of sea lamprey, *Petromyzon marinus*. (a) The full view of the setup, (b) enlarged view of an adult sea lamprey attached to the sensing panel with its mouth covering all the 9 sensing ports, and (c) a schematic of the 9 ports.

Commercial vacuum pressure sensors (Honeywell 40PC0152A) were used to construct the sensing system for monitoring vacuum pressures exerted by sea lampreys on test surfaces. Each pressure sensor had an operating pressure range of 0 kPa to –103.4 kPa and response time of 1 ms maximum. Two types of pressure-sensing apparatuses were developed for comprehensive experiments, including a panel with a single sensing port (**Fig 1**), and a panel with a 9-port circular pressure sensing matrix (**Fig 2**), respectively. The single port system was comprised of an elbow-shape tight-seal moisture-resistant barbed tube connector, which was the smallest we could find to build this pressure sensing system, glued into a 2.5-mm port in a smooth acrylic plate. A soft plastic tube connected the tube fitting to a vacuum pressure sensor 20 cm away. For encapsulation of the port, a 5-mm-thick layer of polydimethylsiloxane (PDMS, Sylgard-184, Dow Corning) was cast on the acrylic plate with a mixing ratio of 10:1 (PDMS base: curing agent, wt.%), and then cured at 70°C for 2 hours. The acrylic plate was then placed vertically in water against the wall of a 200 L aquarium so that the side of the panel with barb fitting, tubing and PMDS was against the glass. During each test, a sea lamprey was gently held with its mouth centered over the port on the acrylic plate until it attached to the plate via oral suction. Pressure data from the sensor, measured in kPa, were acquired by an Arduino processor board (Arduino Uno for single port sensor; Arduino Mega 2560 for 9-port sensing panel) at a sampling frequency of 200 Hz and stored in a computer. The 9-port system was fabricated in a similar way, but contained a central port surrounded by eight ports arranged in a circular pattern with a radius of 8 mm (**Fig 2**) such that all ports would be covered by the sea lamprey’s oral disc.

### Checking the leakage of the pressure sensing system

**Fig 3:**
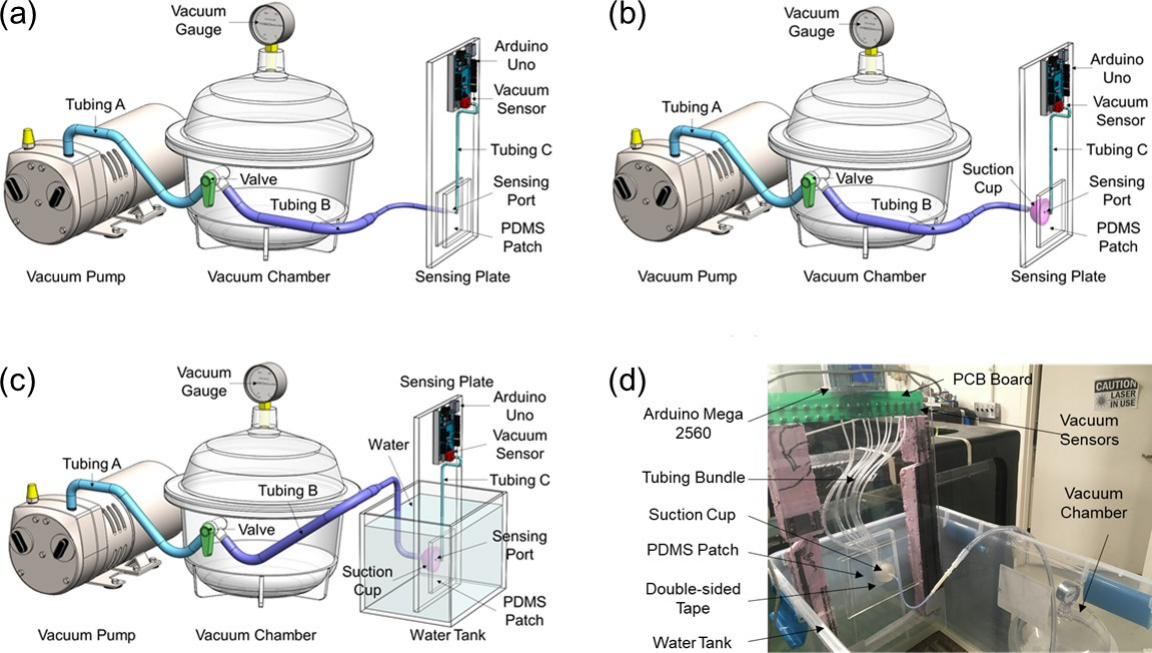
Schematic (not to scale) of experimental setups for checking leakage of the single- and 9-port pressure sensing systems used to measure oral suction pressures of sea lamprey, *Petromyzon marinus*. (a) Apply vacuum from the vacuum chamber to the vacuum sensor through tubing B, sensing port and tubing C, (b) apply vacuum from the vacuum chamber to the vacuum sensor through tubing B, suction cup attached on the plate, sensing port and tubing C, and (c) test setup (d) under water by putting part of the sensing plate and the suction cup under water in a water tank. Abbreviations: PDMS = polydimethylsiloxane; PCB = printed circuit board.

A three-part experiment was used to determine if sea lamprey suction measurements were influenced by air leakage (Objective 1; **Fig 3A–3C**). Specifically, for the single-port pressure sensing system, we used a vacuum pump and vacuum chamber to determine if leakage was related to the type of connection used at the port (direct-fit potted vs. simulated lamprey mouth suction cup), magnitude of suction applied (–10 vs. –20 kPa), or surrounding medium (air vs. water). In the direct-fit test, small tubing from the vacuum chamber was connected to the sensing port on the acrylic plate and PDMS was used to encapsulate the sensing port (**Fig 3A**). The PDMS encapsulation was assumed robust enough that any observed leakage would be attributed to other parts of the system that were also used in measurements of live lampreys (i.e., Tubing B in **Fig 3A**, the vacuum sensor, or the connection between them). A second setup used to simulate oral suction from a lamprey, was comprised of a suction cup (rim diameters: inner, 32 mm, outer, 40 mm; depth: 20 mm) made of PDMS via a molding and casting process, and placed on the sensing port with its inlet connected to the vacuum chamber (**Fig 3B**). Leakage was evaluated as described for the first setup. Finally, to simulate the lamprey’s suction under water, the suction cup setup was immersed in water in a 45 cm x 30 cm x 30 cm water tank (**Fig 3C**). During each test, the vacuum pump was turned on to create vacuum pressure in the vacuum chamber. When the pressure reached the setpoint, a valve on the vacuum chamber was closed to block the inlet between the vacuum pump and the vacuum chamber, but equalize the air pressure between the vacuum chamber and the vacuum sensor. Under this state, the pressure sensor output was recorded for 1000 s and possible leakage was evaluated. For each setup, three times of leakage checking tests, each one lasting for 1000 s, were conducted in order to show the reliability of the single-port pressure sensing system. Similarly, the 9-port pressure sensing system was also tested to check possible leakage under these three setups, with three experiments conducted for 1000 s for each setup. **Fig 3D** shows the counterpart of the direct-fit potting setup for the 9-port sensing system, where a 1.5 mm thick layer of 3M VHB4905 double-sided tape was used to bond the bottom surface of suction cup and the acrylic plate around the nine ports, which had a good seal and any observed leakage would be attributed to other parts of the system.

## Measuring sea lamprey suction dynamics

### Experimental animals

Three experiments were conducted in 2019 and 2020 using spawning phase adult and parasitic juvenile sea lampreys (see **Table 1**). During Experiment 1, suction pressures of adult sea lampreys were measured using the single port pressure sensing system in a 200 L aquarium with static water. During Experiment 2, suction pressures of adult and juvenile sea lampreys were measured using single and 9-port pressure sensing systems in a 200 L aquarium with static water. During Experiment 3, suction pressures of adult sea lampreys were measured using a 9-port pressure sensing system in a 1000 L aquarium with static and flowing water. Adult sea lampreys used in this study were captured in traps during upstream spawning migration in the St. Marys River (Michigan, USA and Ontario, Canada) during July 2019 and 2020. Traps were operated by Canada Department of Fisheries and Oceans and the U. S. Fish and Wildlife Service. Juvenile sea lampreys were collected by commercial fishers in northern Lake Huron during September 2019. All lampreys were transported to the U. S. Geological Survey Great Lakes Science Center’s Hammond Bay Biological Station, Millersburg, Michigan, USA where they were held in aerated 1000 L tanks supplied continuously with Lake Huron water maintained at 8°C until tests were conducted. Prior to tests, body weight, total body length, and mouth diameter were measured for most, but not all sea lampreys (**Table 1**). All sea lamprey experiments were performed in accordance with protocols and guidelines approved by Michigan State University’s Institutional Animal Care and Use Committee (IACUC, No. 02/18-028-00). After the suction pressure experiments in this study, the sea lampreys were housed for use in further research by Hammond Bay Biological Station.

**Table 1:** Summary of vacuum pressure tests of live sea lampreys, including months, instruments used (sensor type), flow conditions (water flow), and biological variables (life stage, sex (M = male; F = female; N/A = unavailable), number of individuals (N), body weight, body length, and mouth diameter).

Experiment	Month	Sensor Type	Water Flow	Life Stage	Sex	N	Body Weight (g)	Body Length (mm)	Mouth Diameter (mm)
**1**	**Aug. 2019**	Single	Static	Adult	M	2	246.5 ± 25.5	490 ± 20.0	N/A
					F	2	233 ± 50.0	480 ± 30.0	32 ± 1.0
**2**	**Oct. 2019**	Single	Static	Adult	M	2	150.0 ± 31.0	416.0 ± 35.0	31.5 ± 1.5
					F	7	224.2 ± 24.8	469.8 ±17.4	32.1 ± 0.9
		9-port	Static	Adult	F	5	198 ± 30.2	459.6 ± 20.8	30.6 ± 2.0
				Juvenile	N/A	6	109 ± 7.4	422.3 ± 11.8	30.5 ± 1.1
**3**	**Aug. 2020**	9-port	Flowing	Adult	M	3	187 ± 34.2	433 ± 31.8	31.3 ± 1.9
					F	4	212.3 ± 26.0	447.5 ± 18.4	27.8 ± 1.4
	**Sept. 2020**	9-port	Flowing	Adult	M	4	147.5 ±17.6	395 ± 16.6	28.2 ± 1.5
					F	5	163.4 ± 16.2	420.0 ± 25.7	25.1 ± 1.6

Data are presented in the type of mean ± standard deviation. Sex could not be determined for juvenile sea lampreys. Mouth diameter was not measured during tests conducted in Aug. 2019.

**Fig 4:**
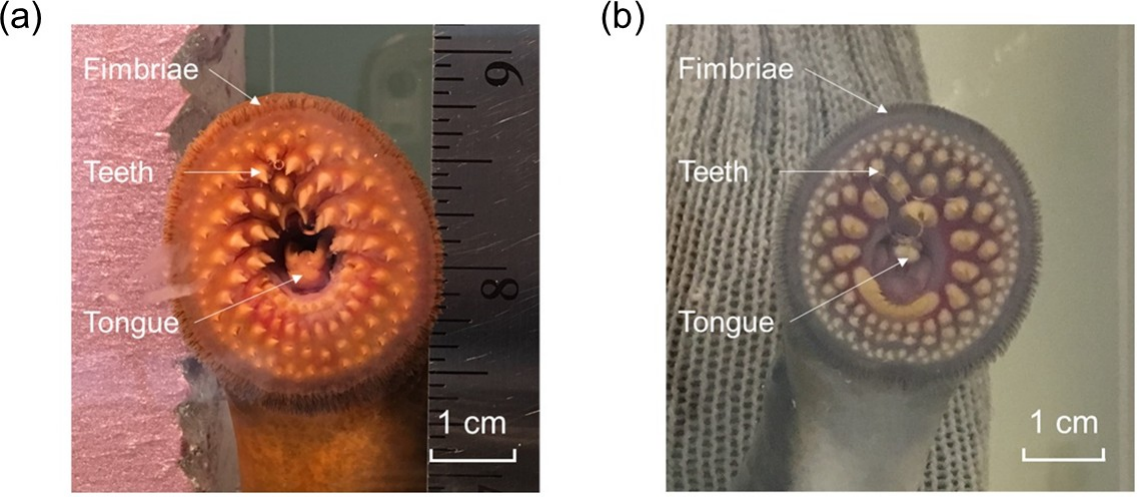
Pictures of the suctorial mouths of the adult and juvenile sea lampreys. (a) Suctorial mouths of an adult sea lamprey, and (b) a juvenile sea lamprey showing that the two are morphologically similar.

**Fig 5:**
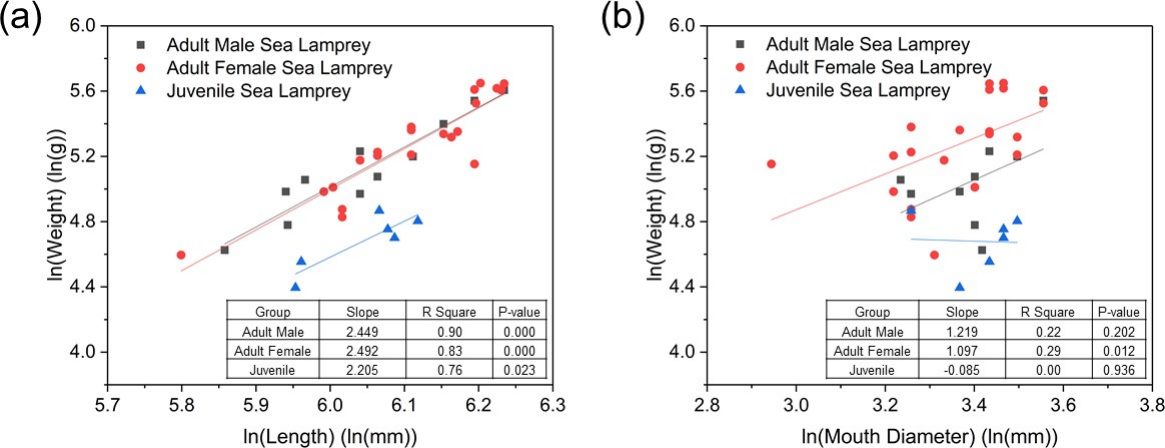
Relations between (a) ln(weight) and ln(length), and (b) ln(weight) and ln(mouth diameter) for tested adult male, female and juvenile sea lampreys.

Juvenile lampreys were shorter and lighter than the adult lampreys on average, less than half of the total weight of adults, but the lampreys of both these two stages had suctorial mouths in similar size and anatomy (e.g., number and location of teeth; **Fig 4**). The natural logarithm (ln) of body weight was positively correlated with the natural logarithms of length of adult male, female and juvenile lampreys (**Fig 5A**). The length-weight relation was similar between male (linear regression: slope = 2.449; R^2^ = 0.90; p-value = 0.000) and female (slope = 2.492; R^2^ = 0.83; p-value = 0.000) adult lampreys, but juveniles (slope = 2.205; R^2^ = 0.76; p-value = 0.023) weighed less than similar-body-length adults, possibly because their reproductive organs were not yet developed. Smaller slope for juveniles than adults may be a consequence of physiological differences between life stages—juveniles are growing while non-feeding adults are senescing(and shrinking). Male (**Fig 5B**; linear regression: slope = 1.219; R^2^ = 0.22; p-value = 0.202) and female (slope = 1.097; R^2^ = 0.29; p-value = 0.012) adult lampreys weighed more than juveniles with similar-sized mouths (slope = –0.085; R^2^ = 0.00; p-value = 0.936), but differences were not large enough to allow sex or life stage determination based on mouth diameter. However, we also reiterate that the juveniles used in this study were in the parasitic form, so it is unlikely that these two life stages would co-occur (e.g., in a stream at the same time).

### Experiment 1: Recording of adult sea lamprey suction dynamics with a single pressure sensor in static water

First, the single-port panel was used to measure suction pressures of 2 male and 2 female adult sea lampreys in August 2019, and another 2 male and 7 female adult sea lampreys in October 2019 in a 200 L rectangular aquarium tank supplied with Lake Huron water but with no noticeable flow (i.e., static flow condition; **Table 1**). Each sea lamprey was measured individually. In the experimental water tank, the sensing panel was placed vertically along a glass wall of the tank so that the side with the elbow fitting and tubing was against the wall and the smooth side with sensing port was available to the sea lamprey (**Fig 1B**). The water level in the tank was about 6 cm higher than the sensing port, submerging all the sensing area around the sensing port. An adult sea lamprey was transferred to the tank and allowed to explore the tank until it attached to the tank surface via oral suction. If the lamprey did not attach onto the sensing area, it would be gently repositioned and held with its mouth over the sensing port until it attached. Pressure measurements were recorded until the lamprey voluntarily detached from the panel or first 20 minutes of attachment elapsed. If a lamprey voluntarily detached or was manually disengaged from the panel after 20 minutes of attachment, it was allowed to rest for 10 min before it was manually reattached to the panel for the pull test. After reattaching to the panel for 5 minutes, the lamprey was gradually detached from the panel by gently pulling the lamprey away from the panel until disengagement. Suction dynamics were summarized from recorded data. If the lamprey detached voluntarily before 5 minutes of static attachment achieved, the lamprey was removed from the tank and manual pull test was not conducted.

### Experiment 2: Recording of juvenile and adult sea lamprey suction dynamics with a 9-port pressure sensing system in static water

A second set of tests were conducted in October 2019 using the 9-port pressure sensing system to determine if suction pressures varied spatially across a sea lamprey mouth (Objective 3). Data were collected from 5 adult female lampreys and 6 juvenile lampreys using the 9-port panel in rectangular aquarium tanks as described above (**Table 1**). No male sea lampreys were used for those tests because they were not available. Adult female lampreys were smaller during October 2019 tests than those measured during August 2019 tests (**Table 1**). Loss of weight between August and October was expected because adult sea lampreys permanently cease feeding prior to commencement of spawning migration and thus lose energy and mass until death occurs. Body sizes of juvenile lampreys were also smaller than adults in October 2019, but suctorial mouths of adults and juvenile were similar in anatomy (e.g., number and location of teeth) and size (**Fig 4**).

### Experiment 3: Recording of juvenile and adult sea lamprey suction dynamics with a 9-port pressure sensing system in flowing water

**Fig 6:**
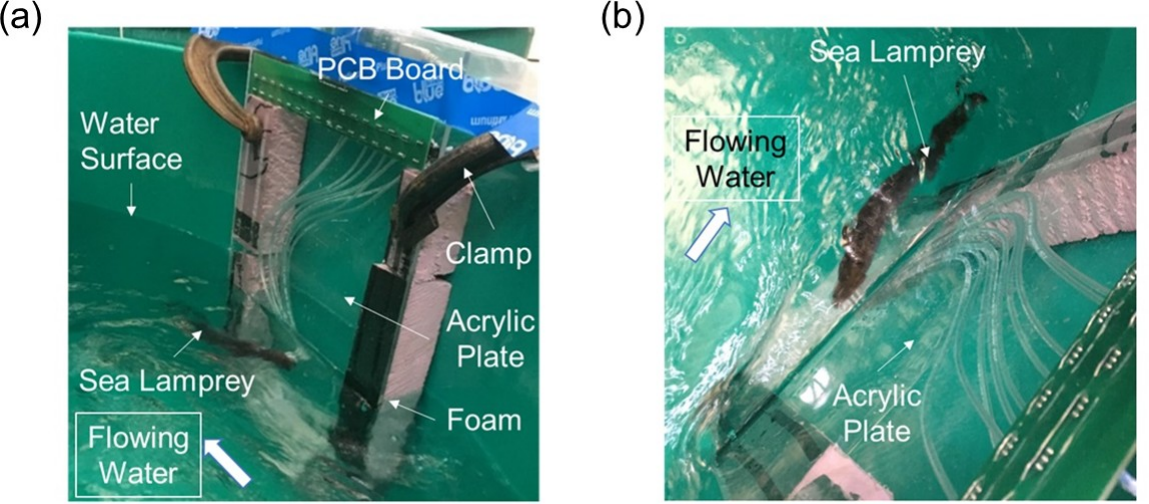
Pictures of experimental setup for recording oral suction pressures of sea lampreys, *Petromyzon marinus*, in flowing water. (a) Side view, and (b) top view. Abbreviations: PCB = printed circuit board.

A third set of tests were conducted in August and September 2020 to determine if suction dynamics differed when the sea lamprey was in static vs. flowing water (Objective 5). In natural aquatic environments like rivers and streams, sea lampreys attach onto rocks or other surfaces for resting; it is of interest to characterize the suction pressure in the presence of water currents. Data were collected from 16 adult lampreys (**Table 1**) using the 9-port panel in a 1000 L circular water tank (1.8 m in diameter) fed continuously with fresh lake water from a spray bar near the surface to create consistent annular water flow around a central stand-pipe drain. The 9-port sensing panel was affixed to the wall of the tank near the surface (**Fig 6**) and water velocity was measured at the sensor panel using a portable flow meter (Marsh McBirney Flo-mate 2000). At the start of each test, each sea lamprey was manually placed on the sensing panel with its mouth over the 9 sensing ports. Each lamprey was tested at four water velocities during each test. The test began with static water conditions for 30 s after which time the water velocity was increased to 0.15 m/s, 0.30 m/s, and 0.45 m/s for 20 s at each velocity. Water flow was then returned to static conditions and the sea lamprey was gently removed. Water velocities tested were within typical ranges in sea lamprey spawning habitats [36, 37].

### Data analysis

Objectives 1 and 2 were addressed using suction pressure measurements from all experiments. For each individual sea lamprey, maximum observed suction pressure, minimum observed suction pressure, and median leakage time were calculated during each test. Non-parametric Mann-Whitney U Tests were used to test the null hypothesis that maximum free suction pressure (in static or flowing water without being manually pulled) and median leakage time did not differ between life stages (juvenile vs. adult; sexes combined for adults) or between sexes (male vs. female; adult life stage only). Test statistics and p-values were calculated according to [38]. Simple linear regressions were used to determine if maximum suction pressure were related to body weight within each group (i.e., adult male, adult female, juvenile). Linear regressions were also used to determine if leakage time was related to maximum suction pressure within each group. Coefficient of determination (R^2^) was used as an indicator of strength of association between independent (e.g., body weight) and response variables (e.g., max. suction pressure). Two-sided T tests were used to test the null hypothesis that the slope between independent and dependent variables was not equal to zero. Regression analysis were conducted using the Regression Analysis Tool in Microsoft Excel 2008. Objective 3 and 4 were addressed using visual assessment of measured pressure curves for each individual sea lamprey during each test.

## Results

### Pressure data for leakage check of the apparatus

**Fig 7:**
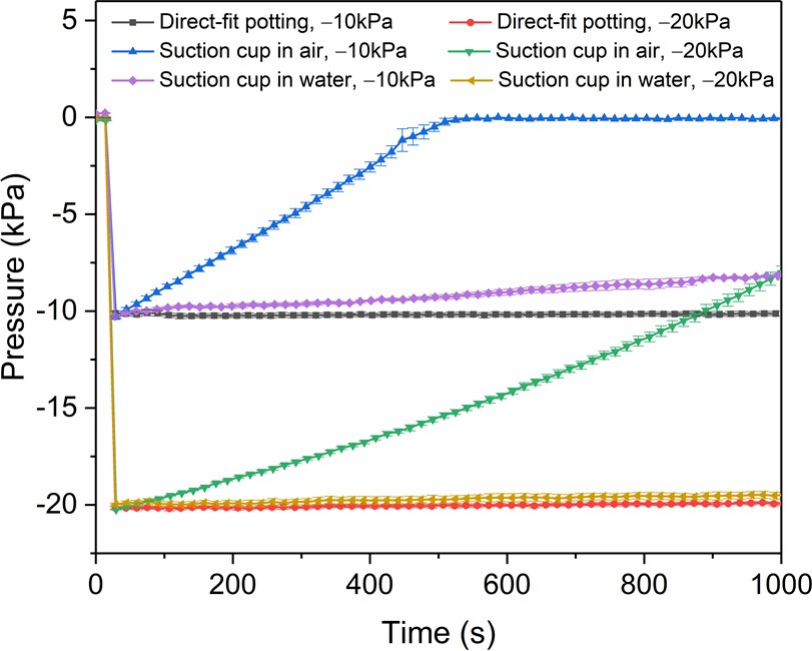
Vacuum pressures recorded during the first 1000 s for potential leakage tests of the single-port pressure sensing setups. Low (–10 kPa) and high (–20 kPa) vacuum pressures were applied with the vacuum chamber tubing attached to the sensor port via one of three methods: direct-fit potting, tested in air (Setup in **Fig 3A**); suction cup interface in air (Setup in **Fig 3B**), suction cup interface in water (Setup in **Fig 3C**). Data were down-sampled for display.

**Fig 8:**
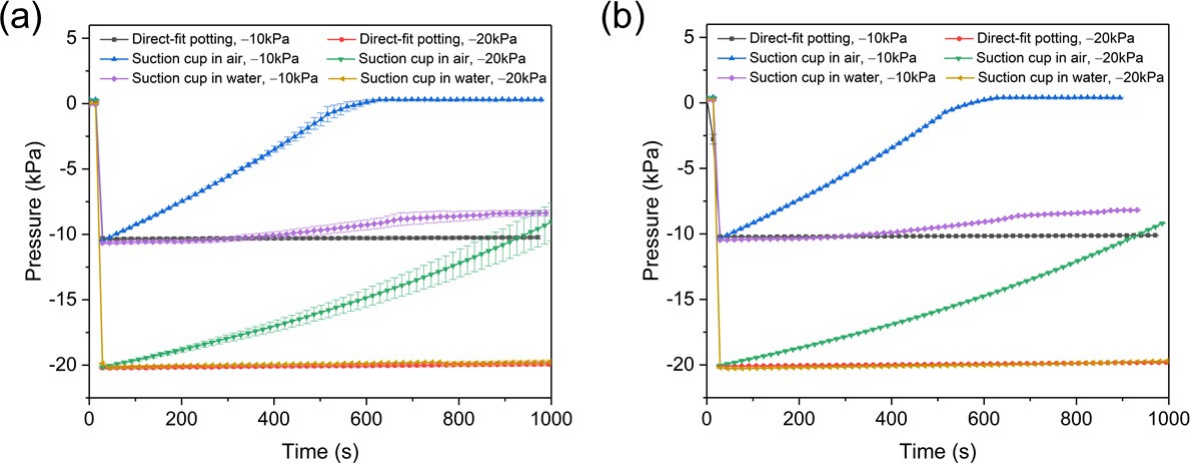
Vacuum pressures recorded during the first 1000 s for potential leakage tests of the 9-port pressure sensing setups. (a) Mean and standard deviation (shown as the error bar) of the pressure at the central port P4 at each time instant for three rounds of measurements, and (b) mean and standard deviation of the mean pressure at all these 9 ports at each time instant for three rounds of measurements.

For the single-port sensing system, when Tubing B was potted directly to the sensing port (**Fig 3A**), initial pressure was maintained for the entire period recorded (1000 s) when initial pressures were –10 kPa and –20 kPa (**Fig 7**), which implies that the system was well sealed. When Tubing B was connected to the sensing port via PDMS suction cup in air (**Fig 3B**), measured vacuum pressure decreased over time, reaching 0 kPa after 500 s with an initial pressure of –10 kPa and reaching about –8 kPa after 1000 s when initial pressure was –20 kPa (**Fig 7**). Slower leakage at higher pressures were attributed to the larger compression of rim of the suction cup (and thus better seal at the interface) under the higher pressure. Finally, the suction cup demonstrated better seal properties when submerged in water. When the suction cup interface was submerged in water, measured pressure decreased to –8 kPa after 1000 s when initial pressure was –10 kPa, and remained near –20 kPa after 1000 s when initial pressure was –20 kPa. Similar results were observed from the 9-port pressure sensing system (**Fig 8**). Measurements from the central port (P4, **Fig 2C**) of the 9-port sensor were very similar to single-port sensor measurements for each connection method and initial pressure (**Fig 8A**). Measurements were also consistent among ports and similar to single port measurements for each connection method and initial pressure (**Fig 8B**). These results indicate that the single-port and 9-port pressure sensing systems (in particular, the connection from the sensing port on the plate to the sensor itself) were well sealed, the reading from the pressure sensor indeed reflected the pressure at the suction point, and that apparent leakage with measurements of live lampreys likely occurred through the interface between the mouth and plate or elsewhere in the lamprey body.

### Suction dynamics of juvenile and adult sea lampreys

**Fig 9:**
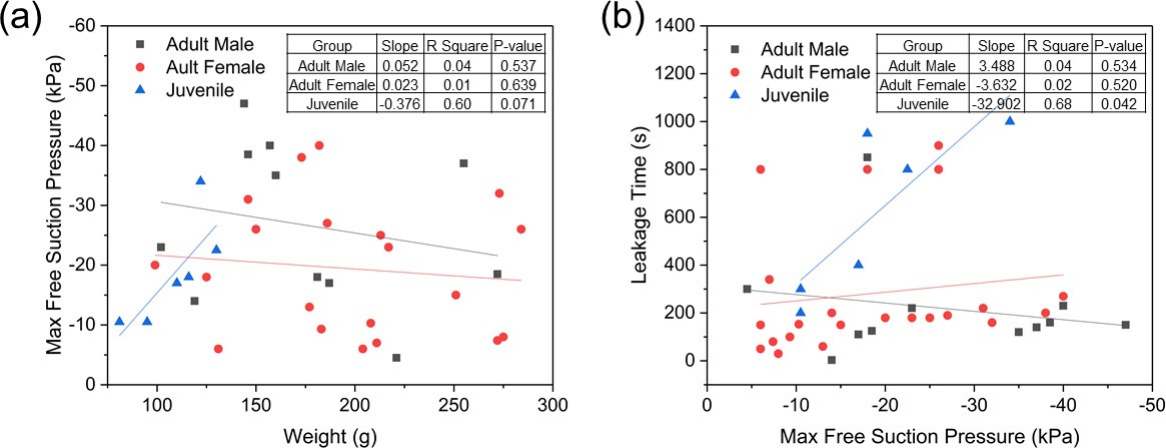
Relations between (a) maximum free suction pressure and weight, and (b) leakage time and maximum free suction pressure for tested adult male, female, and juvenile sea lampreys.

Among all tests using the single-port sensing panel with live adult sea lampreys at rest (excluding pull tests), free suction pressures ranged from –3.3 ± 0.9 kPa to –13.8 ± 3.2 kPa with a leakage time (period between re-pressurizing pumps) of 319.5 ± 187.0 s (**Table 2**). During pull tests, maximum suction pressures ranged from –8 kPa to –70 kPa. Owing to small sample sizes and large variability (**Fig 9**), we did not detect statistically significant differences in maximum free suction pressures between male and female adult sea lampreys (Mann-Whitney U = 77.5, z = –1.342, p-value = 0.180) or between adult and juvenile sea lampreys (U = 80.5, z = –0.515, p-value = 0.607). Similarly, we did not detect differences in median leakage time between male and female adult sea lampreys (U = 104, z = –0.649, p-value = 0.516). However, median leakage time was significantly greater for juvenile than adult sea lampreys (U = 27, z = –2.803, p-value = 0.005). among groups (i.e., adult male, adult female, juvenile). Maximum vacuum pressure was weakly associated with body weight in juvenile sea lampreys (**Fig 9A**; linear regression: slope = –0.376; R^2^ = 0.60; p-value = 0.071) but not adult male (slope = 0.052; R^2^ = 0.04; p-value = 0.537) or female (slope = 0.023; R^2^ = 0.01; p-value = 0.639) sea lampreys. Leakage time was weakly associated with maximum vacuum pressure in juvenile sea lampreys (**Fig 9B**; slope = –32.902; R^2^ = 0.68; p-value = 0.042) but not adult male (slope = 3.488; R^2^ = 0.04; p-value = 0.534) or female (slope = –3.632; R^2^ = 0.02; p-value = 0.520) sea lampreys.

**Table 2:** Statistics of the pressure measurement results corresponding to Table 1, including manual pull condition (pull), maximum suction pressure before disengagement (max vacuum), minimum suction pressure to remain attachment (min vacuum), and the duration period between two adjacent pressurizing processes (leakage time).

Experiment	Sensor Type	Water Flow	Life Stage	Sex	Pull	Max Vacuum (kPa)	Min Vacuum (kPa)	Leakage time (s)
**1, 2**	Single	Static	Adult	M	No	–13.8 ± 3.2	–3.3 ± 0.9	319.5 ± 187.0
					Yes	–39 ± 31	0	19 ± 16
				F	No	–13.2 ± 2.9	–2.5 ± 1.1	379.1 ± 134.8
					Yes	–37.2 ± 4	0	16 ± 4.1
**2**	9-port	Static	Adult	F	No	–10.7 ± 2.4	–1.1 ± 0.3	304 ± 131.3
					Yes	–32.8 ± 7.6	0	10.5 ± 2.3
			Juvenile	N/A	No	–18.8 ± 3.6	–5.4 ± 2.4	608.3 ± 142.8
					Yes	–35 ± 1.2	0	7.1 ± 1.5
**3**	9-port	Flowing	Adult	M	No	–34.0 ± 3.9	–17.4 ± 4.5	161.4 ± 17.7
				F	No	–27.7 ± 2.9	–8.2 ± 1.7	182.2 ± 18.6

**Fig 10:**
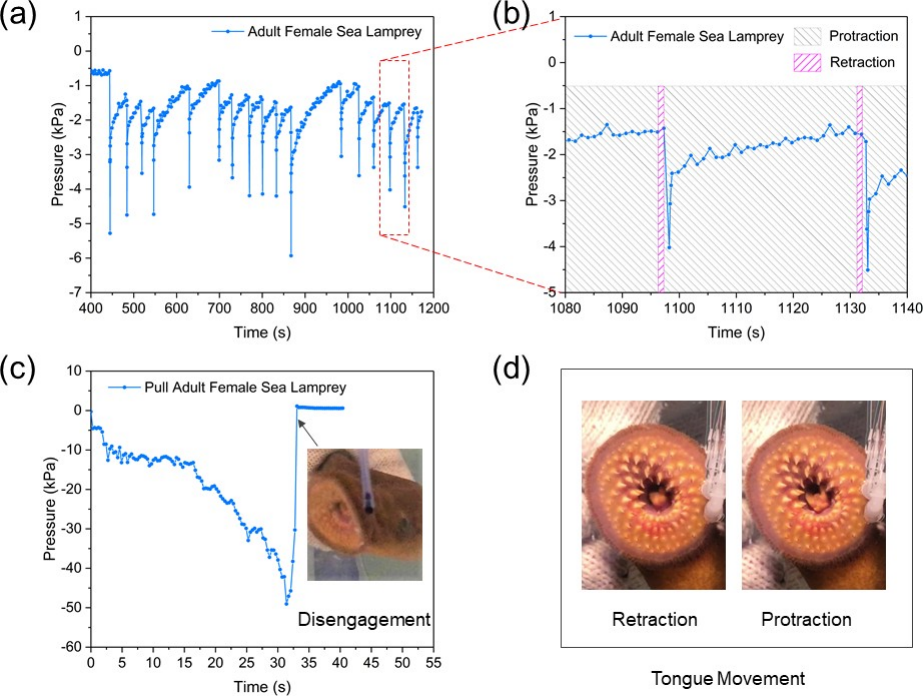
Suction dynamics of an adult female sea lamprey in Experiment 1. (a) Suction pressure when the adult female lamprey attached to the panel during 400 s to 1200 s after attachment, (b) suction pressure profile during 1080 s to 1140 s after attachment with shaded rectangles showing the time when the lamprey’s tongue was protracted or retracted, (c) suction pressure profile when the adult lamprey was pulled away from the panel with corresponding snapshot of the lamprey’s mouth disengaged from the panel, and (d) pictures of an adult lamprey’s mouth showing the protraction and retraction states of its tongue.

Observed suction dynamics were largely consistent with previous descriptions but high measurement rate coupled with experimental video provided detailed insights. For example, during the 20 min experiment of suction on the sensing panel without change of attaching position, the suction pressure of an adult female lamprey frequently fluctuated between –6 kPa and –1 kPa (**Fig 10A**). It rose to –6 kPa suddenly in about 0.3 s, and then slowly returned to –1 kPa in about 30 s. For some cycles, the recovering time lasted for 50~100 s. During static attachment, the lamprey’s tongue was observed to protract (**Fig 10B**; see also **S1 Video**), i.e., the buccal cavity and the pharyngeal cavity were connected due to the protraction of the tongue, and when the suction pressure was too low to maintain attachment, the tongue retracted quickly to enclose the buccal funnel, and then, protracted immediately again to open the oral passage (the timing of these tongue movements is indicated by the shaded rectangles in **Fig 10B**), which was consistent with the suction mechanism described in [24]. With these actions, the suction pressure increased rapidly, maintaining attachment on the panel. Suction pressure leakage was common, and leaking and pressurizing processes alternated until disengagement from the panel.

During the pull test (details in **S2 Video**) of the same adult lamprey suction pressure rose slowly from –5 kPa to –50 kPa in about 30 s, and then returned to 0 kPa in only one second (**Fig 10C**). During this test, the suction pressure increased to 10 times of its initial level, and the sea lamprey’s tongue retracted and protracted more frequently and with larger amplitude (**Fig 10D**). To resist the drag force, the annular muscle around the mouth contracted more vigorously until disengagement.

### Suction pressure distribution within the mouth of adult and juvenile lampreys

Among all tests using the 9-port sensing panel with live adult and juvenile sea lampreys at rest (excluding pull tests), free suction pressures ranged from –0.5 kPa to –34 kPa (**Table 2**). During pull tests, maximum suction pressures ranged from –16 kPa to –47 kPa. The leakage time varied within and among lampreys, but ranged from 80 s to 800 s for adult lampreys and more than 800 s for a half number of the tested juvenile lampreys.

**Fig 11:**
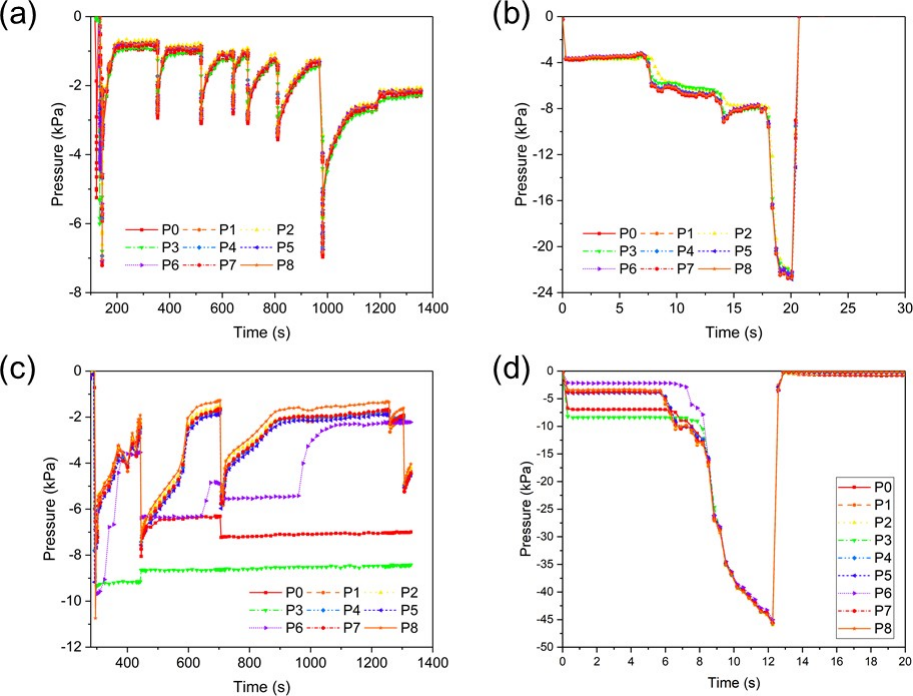
Suction dynamics of two adult sea lampreys in Experiment 2. (a) Suction pressure when an adult female lamprey was attaching by itself on the panel, (b) suction pressure when the lamprey in (a) was pulled away from the panel, (c) suction pressure when another adult female lamprey was attaching by itself on the panel, and (d) suction pressure when the lamprey in (c) was pulled away from the panel.

Suctions pressure measurements with the 9-port sensing system suggested that pressure distributions were uniform throughout the mouth in some cases but not all. For example, suction pressures from an adult female sea lamprey were very similar among all nine sensor ports during resting state and pull tests, despite rapid changes in suction pressure over time (**Fig 11A and 11B**). For that individual, the median leakage time was about 150 s, and the maximum pressure in static suction was about –7 kPa, while the maximum in the pull test was about –22.5 kPa (refer to **S3 Video**). However, suction pressure from another adult female lamprey varied considerably among some sensor ports with no apparent pattern over time or among tests (**Fig 11C and 11D**). For this individual, the median leakage time was about 160 s, and the maximum pressure in static suction was about –10 kPa, while the maximum in the pull test was about –45 kPa. Specifically, the curves representing ports P0, P3 and P6 (labeled in **Fig 2C**), which were located at the left column of the ports, remained at some pressure levels for a few minutes while pressures fluctuated in synchrony among other ports. However, as seen in the picture in **Fig 2B** (which corresponds to this case), these three ports were under the coverage of the oral disc. Hence, this pressure difference might be related to the blocking of the ports by some teeth on the oral disc. It is hypothesized that the oral disc covered the port matrix and formed vacuum pressure in that area, but then some teeth might have fallen into the ports and blocked the passage between the buccal cavity of the mouth and the tubing connected to the pressure sensor, causing the pressure stagnation.

**Fig 12:**
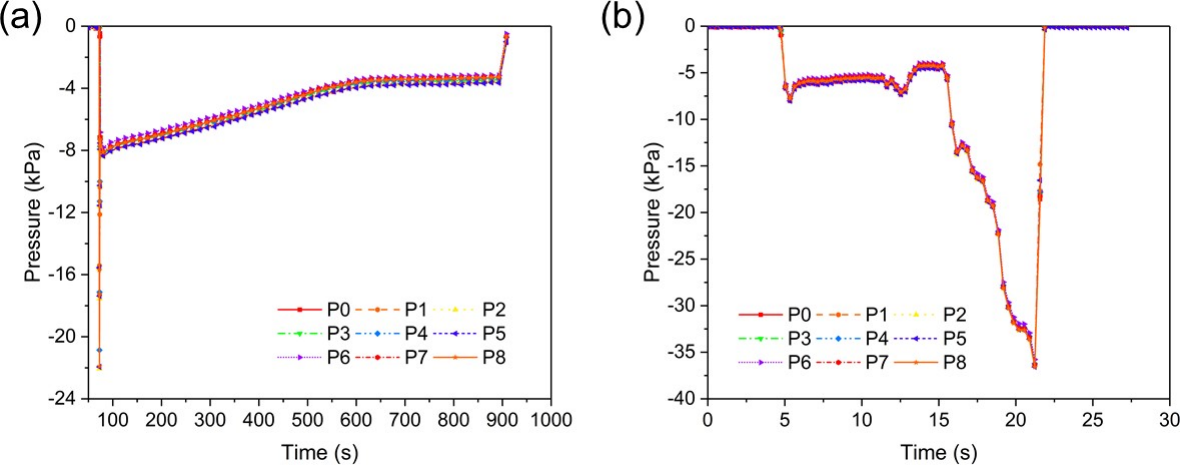
Suction dynamics of a juvenile sea lamprey in Experiment 2. (a) Suction pressure when a juvenile lamprey was attaching by itself on the panel, (b) suction pressure when the lamprey in (a) was pulled away from the panel.

As a final example, suction pressures from one juvenile lamprey were very consistent among all the sensor ports during static suction and pull tests (**Fig 12A and 12B**). The juvenile lamprey created a maximum suction pressure of –22 kPa and hence an effective –8 kPa in the static states, and maintained attachment without pressurization for about 850 s. From the pull test, it was recorded that the maximum suction pressure reached –37 kPa. And the suction pressure profiles of other juvenile sea lampreys tested in this experiment showed similar pressure range and duration time of attachment to this example case both at rest and in pull tests. Besides, compared to the pressure profiles of those adult female lampreys (**Fig 11A–11D**), the juvenile lampreys appeared to be physically stronger as they created higher maximum suction pressure at rest, and each period of attachment without pressurization lasted longer than the adult lampreys.

### Effects of flowing water on measured suction pressure

Among all tests in August and September 2020 using the 9-port sensing panel with live adult lampreys in both static and flowing water (excluding pull tests), suction pressures ranged from –2.5 kPa to –40 kPa (**Table 2**), the maximum of which was much higher than that of the static water tests in August and October 2019. The median leakage time varied within and among lampreys, but were all longer than180 s, which implied the pump frequencies were lower than those observed in the tests in 2019. More importantly, as the water velocity increased step by step, the suction pressure of the adult lampreys did not appear to increase accordingly, nor was a new suction event (re-pressurization) detected from the pressure data. In contrast, the suction pressure of adult lampreys seemed to be insensitive to water flows (velocity ≤ 0.45 m/s), and decreased slowly due to water leakage.

**Fig 13:**
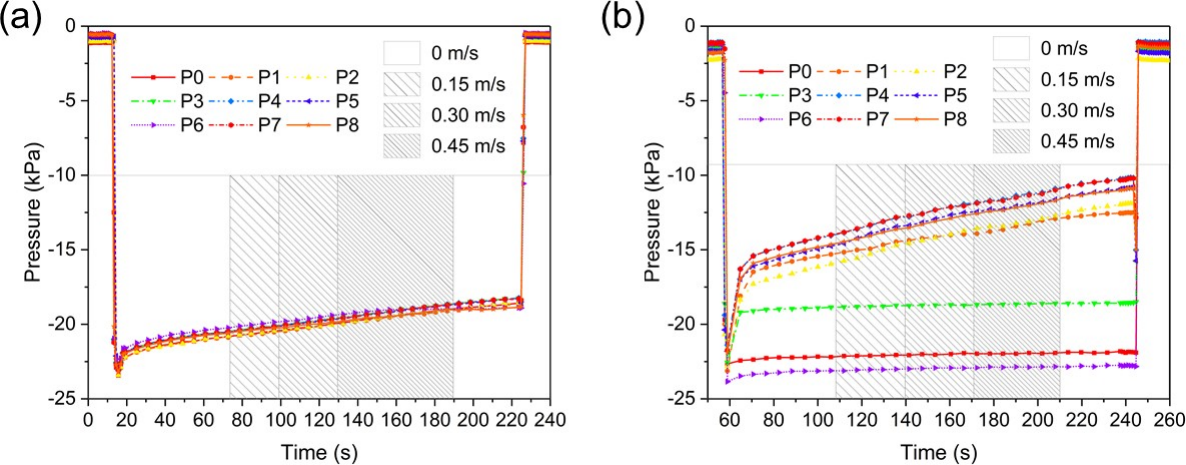
Suction dynamics of two adult sea lampreys showing characteristic responses in Experiment 3. (a) Suction pressures of an adult male lamprey in the flowing water test, and (b) suction pressures of an adult female lamprey in the flowing water test.

For example, suction pressure of an adult male sea lamprey gradually decreased from initial pressure of –23 kPa to –18 kPa while water velocity increased from 0 m/s to 0.45 m/s (190 s) and then was abruptly reduced to 0 m/s where it remained until disengagement at the end of the test (225 s; **Fig 13A**). Pressure curves were similar among all nine sensor ports during the test. Similarly, suction pressure of an adult female lamprey showed no response to changing water velocities during a flowing water test (**Fig 13B).** The rate of leakage, however, did vary among sensor ports during that test, perhaps because three ports were blocked by teeth. Regardless, suction pressure did not increase as water flow increased (≤ 0.45 m/s, see **S1 File** for the suction pressure plots **Fig 1**–**16 in S1 File** of those 16 adult lampreys tested in flowing water, with their tag number, sex, weight, body length and mouth diameter listed in **Table 1 in S1 File**).

## Discussion

We successfully recorded the suction dynamics for two life stages of sea lampreys in static and flowing water. Observed suction behavior was consistent with previously described suction mechanisms [24] and qualitatively similar to previous suction pressure measurements [24, 25], and additional insight obtained may be important for future sensor design and practical deployment. To our knowledge, our measurements are the first to describe suction of adult sea lampreys in flowing water, and to describe variation across the mouth opening. Our 9-port sensor revealed that the pressure distributions were uniform across the lamprey’s oral disc, but that obstruction of sensor ports by teeth may impose measurement error for certain devices. Finally, coupling of video recordings with high-sampling-frequency (200 Hz) pressure measurements revealed the lamprey’s tongue movement and the oral disc contraction during the pull test, which supported previous descriptions of the suction mechanism.

Observed minimum suction pressures showed that lampreys are capable of maintaining attachment to smooth surfaces with relatively little suction, even in flowing water. As shown in **Figs 10A** and **11A**, the suction pressure levels at each moment of the re-pressurization processes were less than about –0.6 kPa, which implied a threshold of pressure level that triggers re-pressurization. When the suction pressure was higher than this threshold, the lamprey did not take action but remained attached; whereas if the leakage reduced the pressure below the threshold, a pressurization process would be taken quickly. The pressure threshold likely depends on a number of variables, including the area and texture of the attachment substrate, size and health condition of the lamprey, the attachment orientation during suction, and the ambient current flow or any other external force causing disengagement.

Although sea lampreys may be capable of attaching to certain surfaces with relatively small suction pressures, leakage at the mouth-substrate interface may necessitate the need for lampreys to apply relatively large suction pressures (e.g., two orders of magnitude greater) at the start of each pump cycle. A trade-off may exist between pump magnitude (pressure applied) and frequency, wherein greater pressure applied to each pump results in fewer pumps overall due to longer time until low-pressure threshold is reached, though we did not detect any association between maximum pressure and leakage time in this study. Results from our leak test further suggest that higher pressure may improve the seal between the oral disk and the substrate, thereby further reducing the leak rate and the number of pumps required per unit time. Indeed, the rate of leakage slowed as pressure decreased in each of our tests. Unlike minimum pressures, however, maximum suction pressures were highly variable among individual lampreys, although it remains unknown if pressure applied is context-dependent, if lampreys can sense the amount of pressure applied, or if pressure applied is simply related to anatomical proportions (e.g., volume of buccal cavity).

Clearly, any device used to detect sea lamprey suction and differentiate it from other sources needs to detect the range of negative pressures exerted by lampreys (described above). To our knowledge, suction dynamics of other organisms and elements of the environment have not been characterized in sea lamprey habitats, though we speculate that that the risk of false-positive measurements from non-lamprey sources, such as water currents, are greater at lower pressures. Thus, a threshold pressure for reliable sea lamprey identification may need to be greater than the minimum pressure observed in sea lampreys. Fortunately, our results imply that negative pressure is uniform throughout the mouth, so a single sensor should be adequate, although improved designs may be needed to prevent obstruction of port holes by teeth. For example, pressure curves in **Fig 11C** where all the nine ports were covered by the oral disc, six pressure curves coincided with each other during the 20 min test, but the other three pressure curves remained at different levels. It was inferred that after the seal of the suctorial disc on the sensing plate, three ports on the left column of the 9-port sensing plate might have been blocked by teeth, which cut off the pressure transduction between the ports and the vacuum sensors. Therefore, the corresponding pressure curves at these three ports remained at some level for a long period without decreasing, while those of the other six ports gradually leaked until a sudden re-pressurization process was induced.

Similarly, the uniformity of measured pressures from juvenile lampreys was due to the characteristics of their teeth, which were smaller and shallower (less than 1 mm long) than the adult lampreys (about 2 mm long in a cone-hook shape). Hence, the juveniles’ teeth would have a lesser chance to block the ports on the panel, and all the ports below the oral disc should be connected to each other and shared the same pressure. Future sensors of the design used in our study may benefit from modifying the port hole size to minimize occlusion by teeth, or compensating the influence by connecting port hole matrix to the same pressure transduction channel.

We also compared the suction pressures of juvenile lampreys (**Fig 12A**) with those of adult lampreys (**Fig 11A and 11C**). Observed differences in suction pressures and leakage times between juvenile and adult sea lampreys may have been related to different energetic states of two life stages. We hypothesize that some juveniles were in general physically stronger than adults at the end phase of the lifecycle, demonstrating higher suction pressures and longer seal, so the juvenile lampreys did not have to re-pressurize as frequently. This might be attributed to the biological activity of the lampreys in different life stages: the juvenile lampreys were in the parasitic stage, and thus were more active and energetic, with stronger attachment for preparation of parasitizing and feeding; the adult lampreys, on the other hand, were presumably much weaker after a long period of non-feeding during the spawning stage, resulting in the suction pressure fluctuating frequently due to leakage. We also analyzed the suction dynamics of adult lampreys in both static and flowing water (**Fig 13**). By increasing the water velocity from 0 to 0.45 m/s step by step while the lamprey was attaching to the sensing panel, we found that the influence of water flow on the suction dynamics of adult sea lampreys was insignificant. Apparently, suction pressure was adequate for maintaining attachment in flowing currents with water velocity below 0.45 m/s.

Oral suction is a multi-function tool that sea lampreys use for feeding, transportation, resting, nest building, and mating, yet has not been utilized for species-specific detection to inform conservation or management. Observations from our new pressure-sensing panels have confirmed previous descriptions of suction dynamics of adult sea lampreys on smooth surfaces and provided new insights into the suction parameters of sea lampreys at the juvenile life stage and in flowing water. Our 9-port sensor array allowed us to investigate the suction dynamics across the lamprey’s oral disc. These results are expected to inform development of the next generation of lamprey assessment gears and may inspire similar efforts to develop detection systems for other taxa with unique characteristics. Finally, sea lampreys often attach onto other living organisms and rocks in the water, but it is still unclear how different attachment surfaces engineered with different materials and roughness patterns will affect the suction behavior. This will be part of our future work when designing deployable sensing panels for operation in natural environments.

## Supporting information

S1 Video Static suction pressure test on an adult female sea lamprey using the single pressure sensor. (MOV)

S2 Video Pull test on an adult female sea lamprey using the single pressure sensor. (MOV)

S3 Video Pull test on an adult female sea lamprey using the 9-port sensing panel. (MOV)

S1 File Suction pressure plots of 16 adult sea lampreys tested in flowing water (with velocity ≤ 0.45 m/s). (DOCX)
