# Supplementary material for: Measurement of suction pressure dynamics of sea lampreys, Petromyzon marinus
Source: PLoS One. 2021 Apr 27;16(4):e0247884. doi: 10.1371/journal.pone.0247884 (PMC8078809; doi:10.1371/journal.pone.0247884)
Supplement: S1 File — (DOCX) [file pone.0247884.s004.docx]

**S1 File. Suction pressure plots of 16 adult sea lampreys tested in flowing water (with velocity ≤ 0.45 m/s).**

**Measurement of Suction Pressure Dynamics of Sea Lampreys, *Petromyzon marinus***

**Hongyang Shi^1¶^, Christopher M. Holbrook^2¶^, Yunqi Cao^3^, Nelson Sepúlveda^1^, Xiaobo Tan^1,^***

^1^Department of Electrical and Computer Engineering, Michigan State University, East Lansing, Michigan, United States of America

^2^U. S. Geological Survey, Great Lakes Science Center, Hammond Bay Biological Station, Millersburg, Michigan, United States of America

^3^College of Control Science and Engineering, Zhejiang University, Hangzhou, Zhejiang, China

*Corresponding author

E-mail: xbtan@egr.msu.edu (XT)

^¶^These authors contributed equally to this work.

**Table 1. Summary of the biological information of the live adult sea lampreys tested in flowing water (with velocity ≤ 0.45 m/s) using the 9-port sensing system in August and October 2020, including pin tag number, sex, body weight, body length and mouth diameter.**

| **NO.** | **Tag** | **Sex** | **Weight (g)** | **Length (cm)** | **Mouth Diameter (mm)** |
| --- | --- | --- | --- | --- | --- |
| 1 | Blue 001 | M | 255 | 49 | 35 |
| 2 | Blue 013 | M | 160 | 43 | 30 |
| 3 | Pink 039 | F | 213 | 45 | 29 |
| 4 | Orange 047 | F | 217 | 45 | 26 |
| 5 | White 042 | M | 146 | 38 | 29 |
| 6 | Orange 024 | F | 273 | 49 | 31 |
| 7 | Pink 042 | F | 146 | 40 | 25 |
| 8 | Blue 021 | M | 187 | 42 | 31 |
| 9 | Pink 038 | F | 177 | 42 | 26 |
| 10 | Blue 049 | M | 102 | 35 | 26 |
| 11 | White 050 | M | 157 | 39 | 30.5 |
| 12 | Blue 045 | F | 99 | 33 | 19 |
| 13 | Pink 010 | F | 186 | 43 | 25 |
| 14 | Pink 006 | M | 144 | 42 | 25.4 |
| 15 | Pink 031 | F | 173 | 49 | 28 |
| 16 | Blue 043 | F | 182 | 43 | 27.4 |


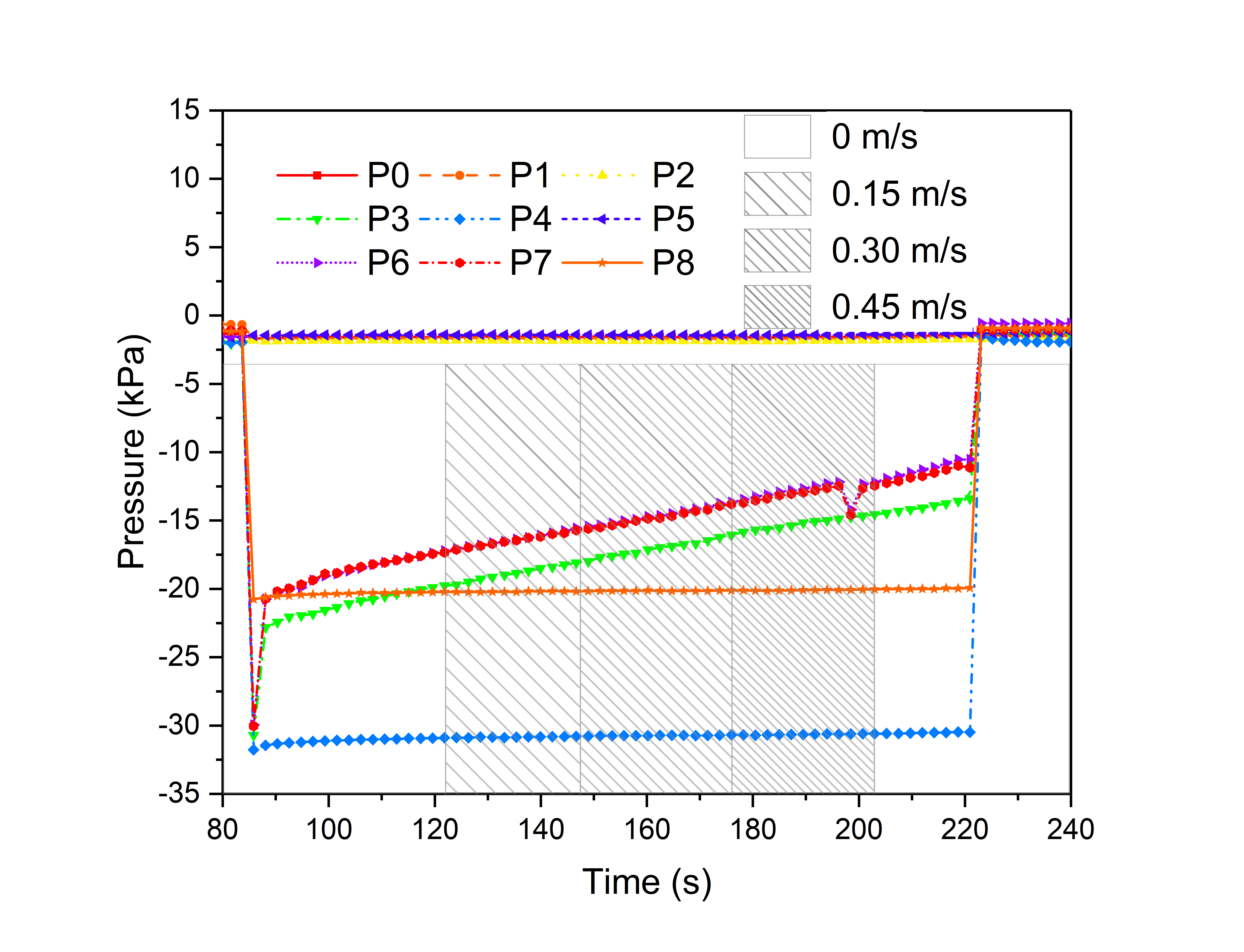


**S4-Fig 1.** **Suction dynamics of the adult male lamprey tagged as Blue 001 in Experiment 3.**


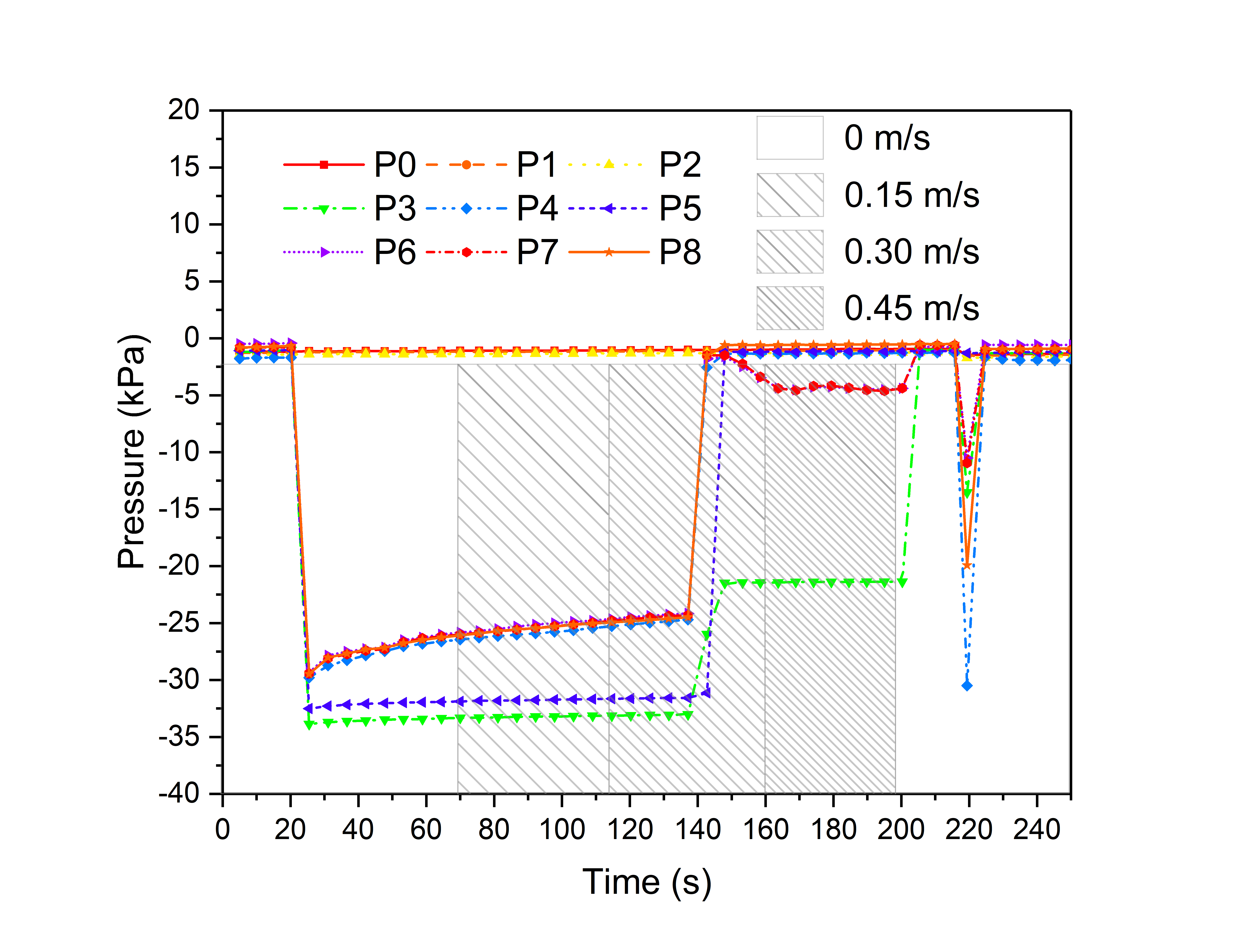


**S4-Fig 2.** **Suction dynamics of the adult male lamprey tagged as Blue 013 in Experiment 3.**


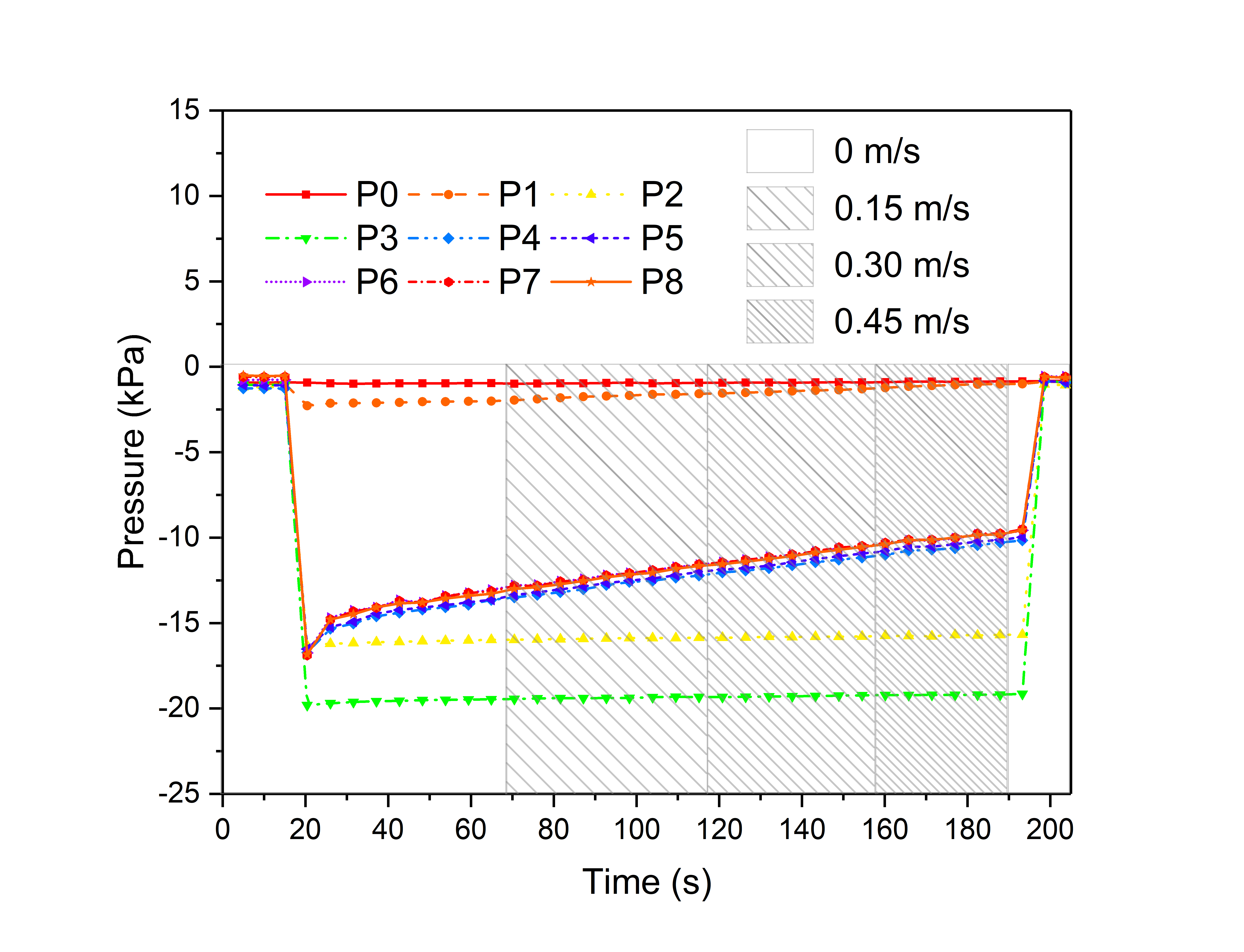


**S4-Fig 3.** **Suction dynamics of the adult male lamprey tagged as Pink 039 in Experiment 3.**


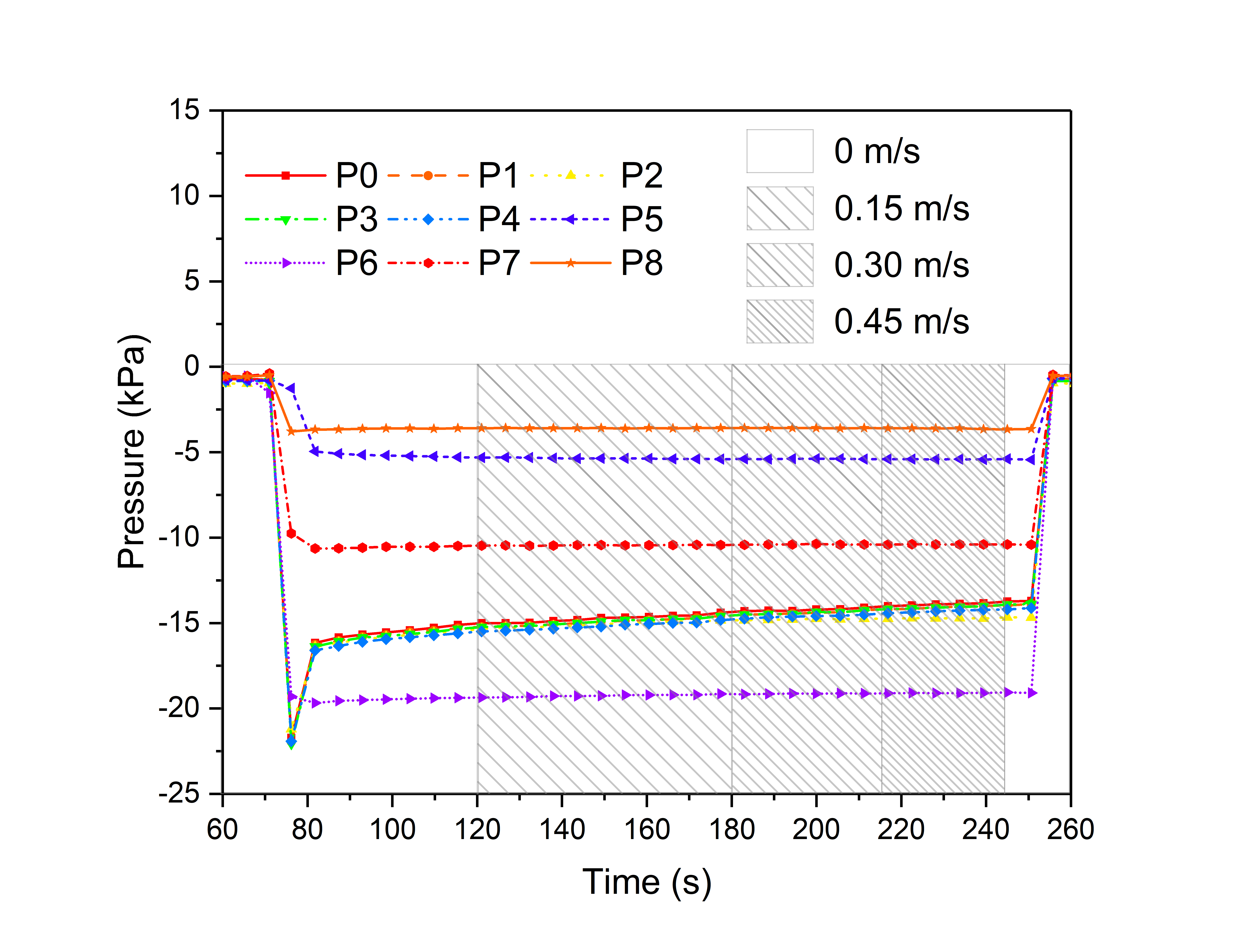


**S4-Fig 4.** **Suction dynamics of the adult male lamprey tagged as Orange 047 in Experiment 3.**


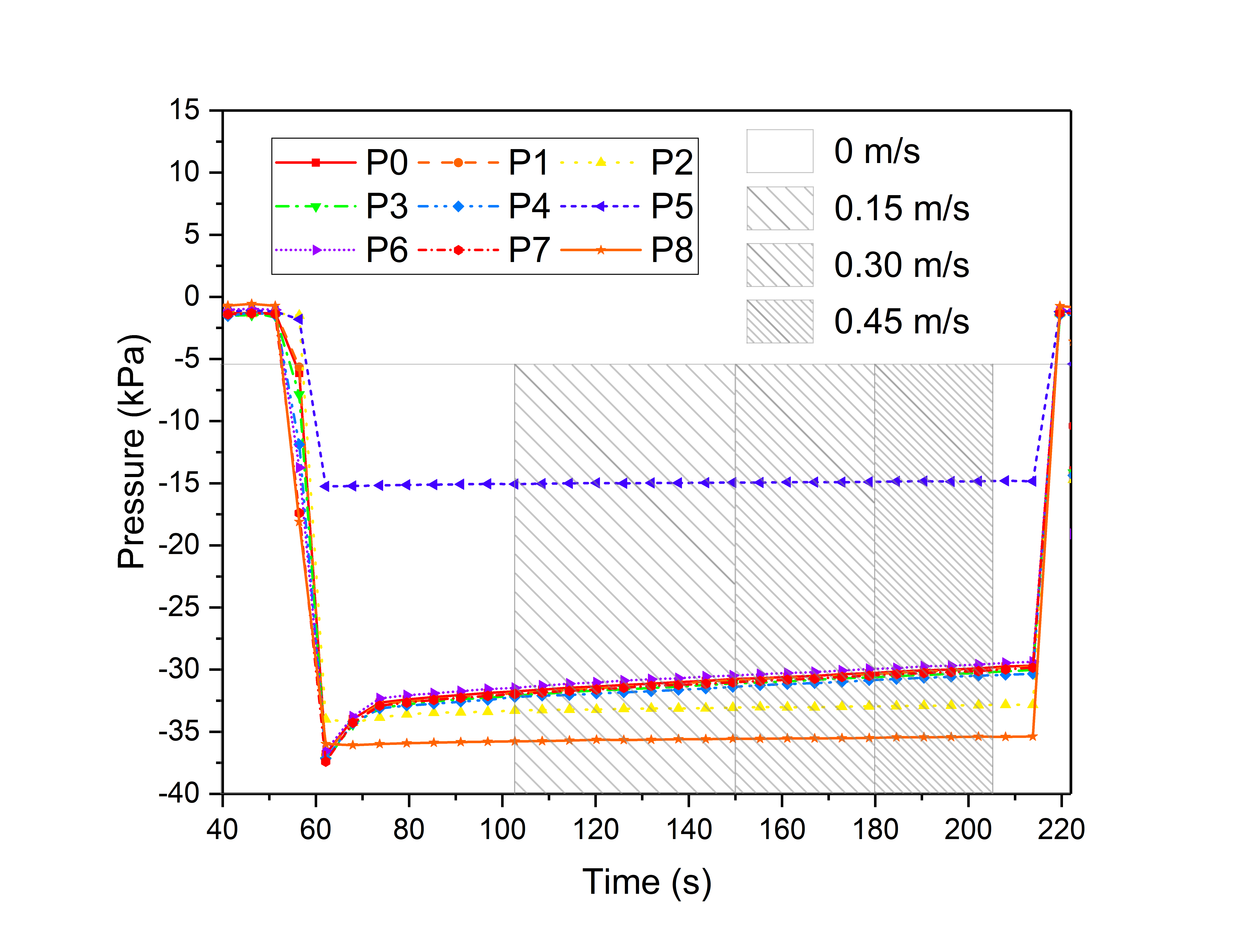


**S4-Fig 5.** **Suction dynamics of the adult male lamprey tagged as White 042 in Experiment 3.**


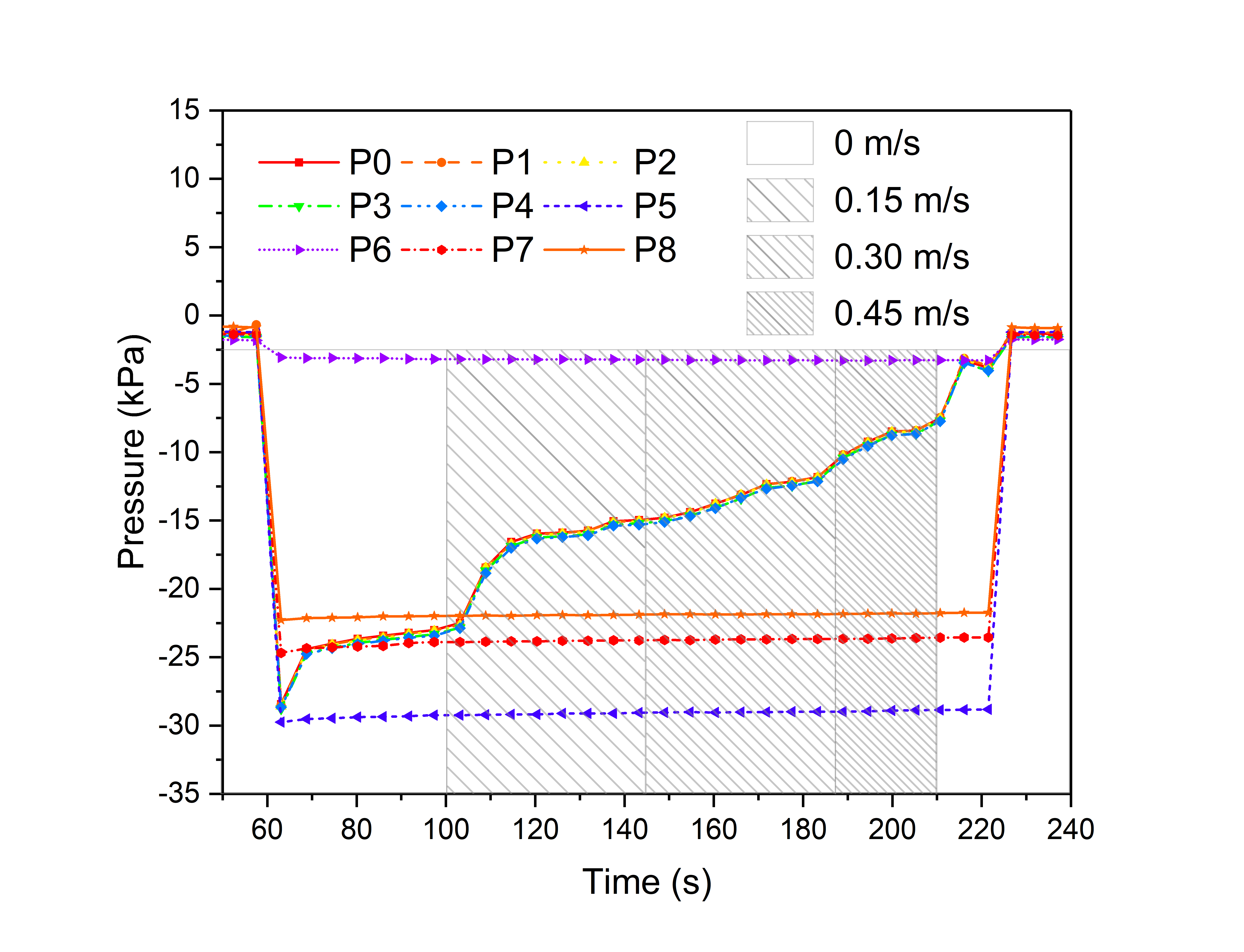


**S4-Fig 6.** **Suction dynamics of the adult male lamprey tagged as Orange 024 in Experiment 3.**


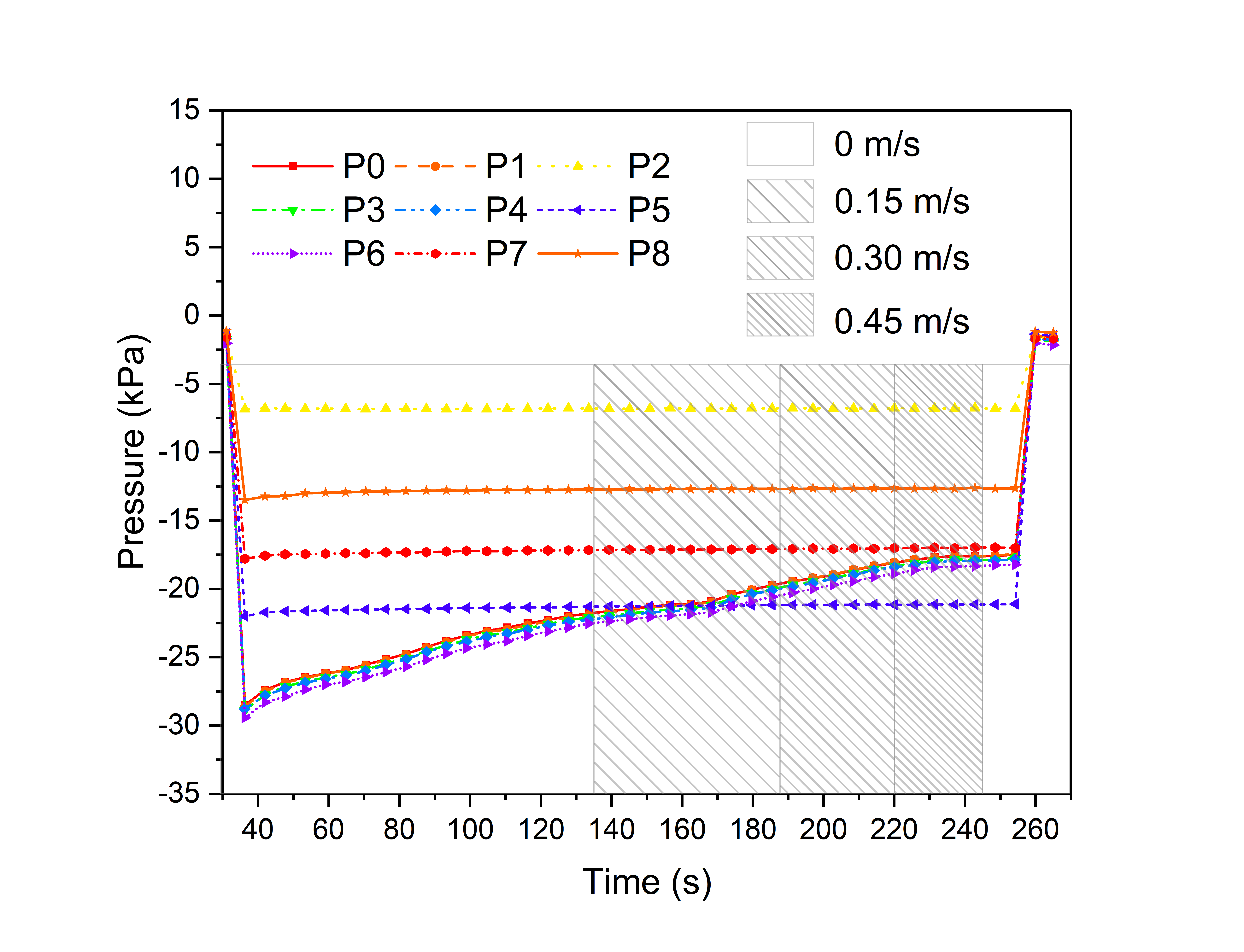


**S4-Fig 7.** **Suction dynamics of the adult male lamprey tagged as Pink 042 in Experiment 3.**


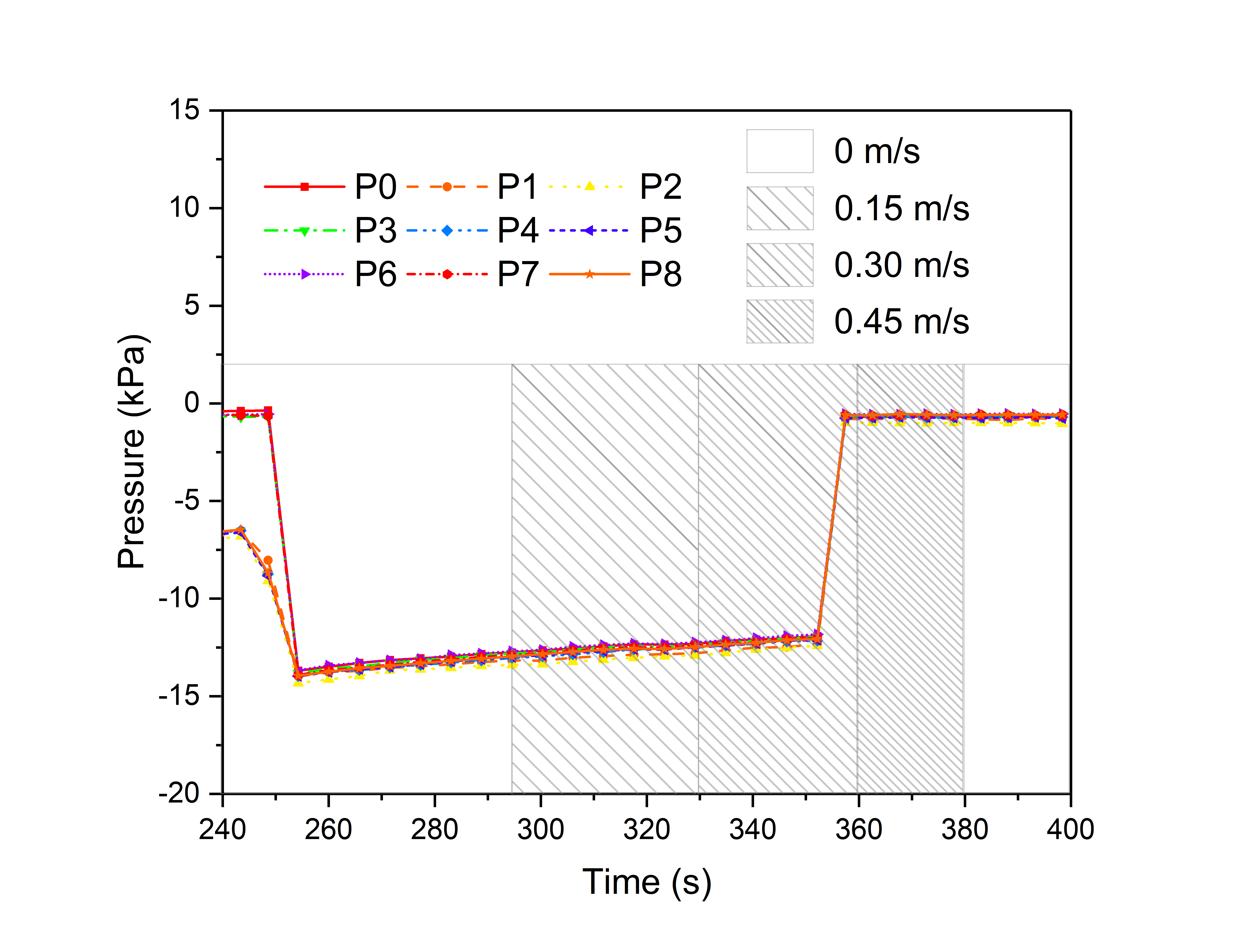


**S4-Fig 8.** **Suction dynamics of the adult male lamprey tagged as Blue 021 in Experiment 3.**


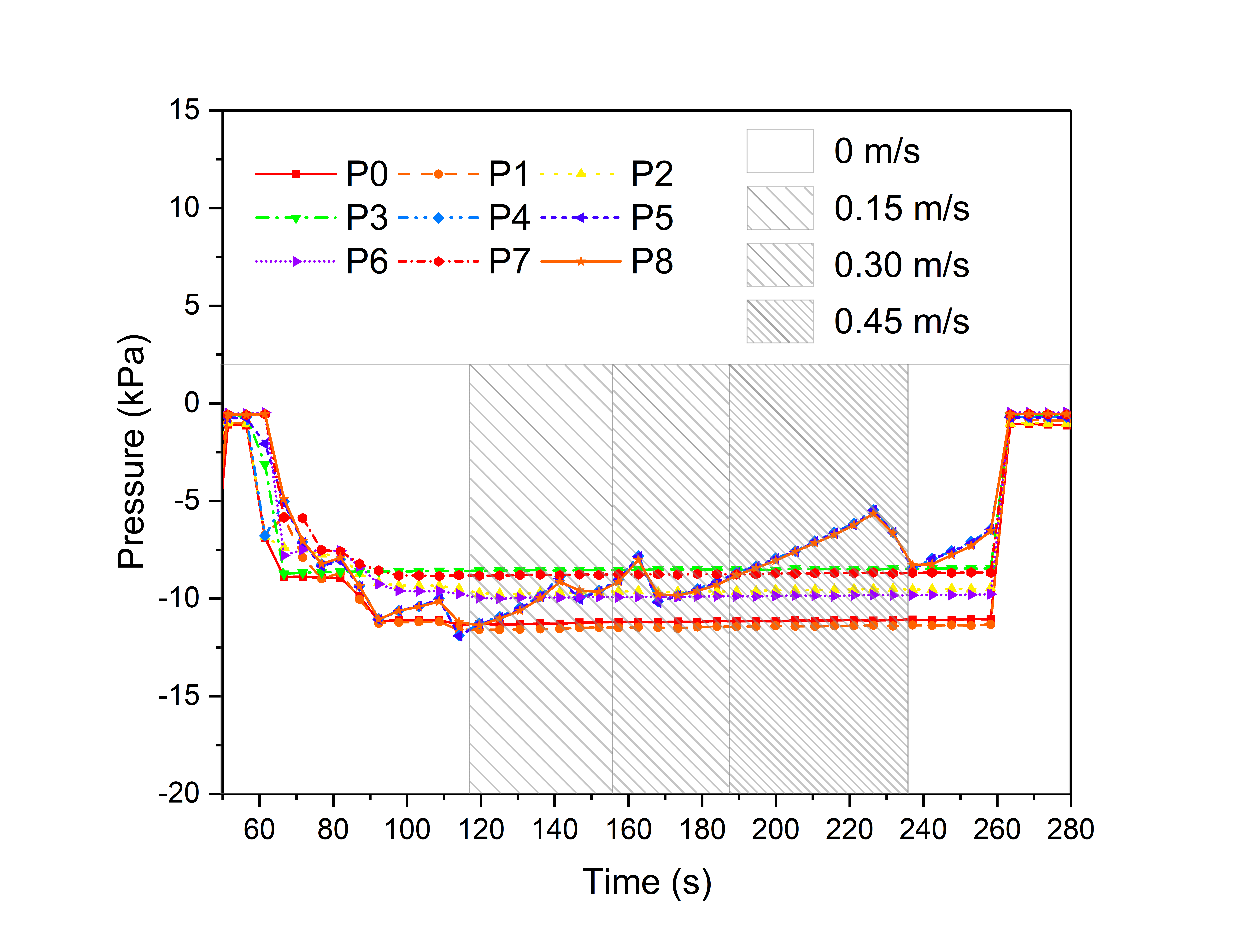


**S4-Fig 9.** **Suction dynamics of the adult male lamprey tagged as Pink 038 in Experiment 3.**


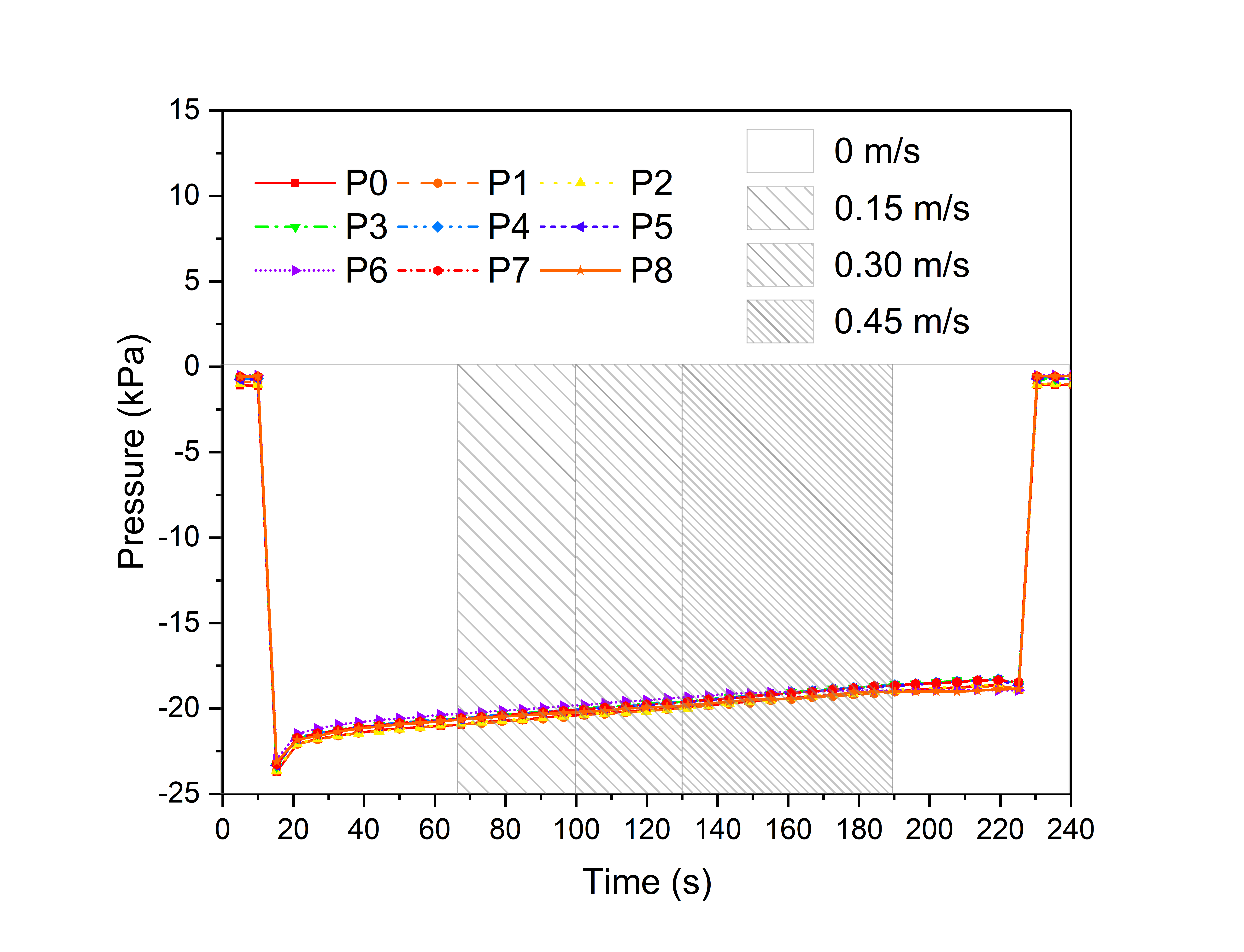


**S4-Fig 10.** **Suction dynamics of the adult male lamprey tagged as Blue 049 in Experiment 3.**


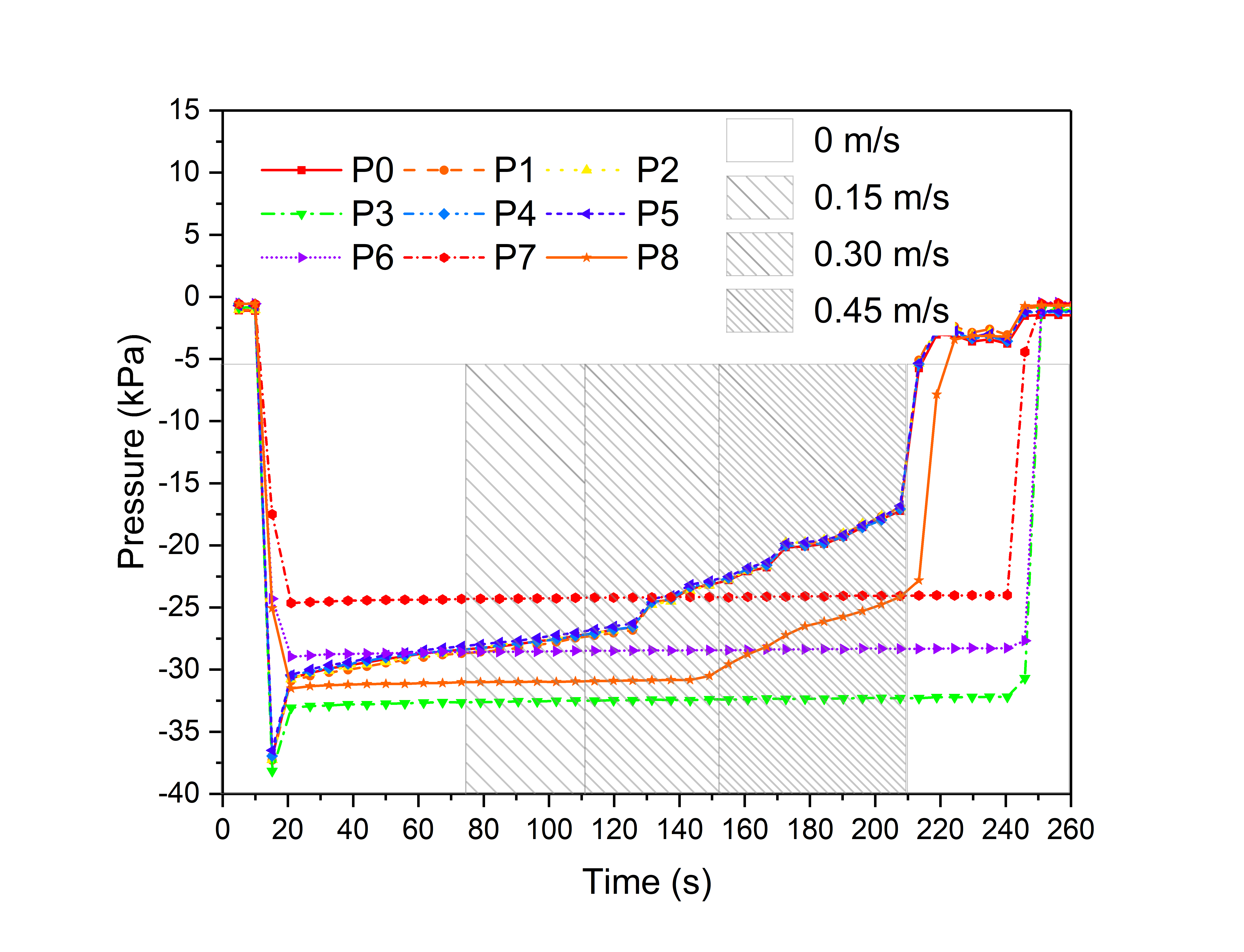


**S4-Fig 11.** **Suction dynamics of the adult male lamprey tagged as White 050 in Experiment 3.**


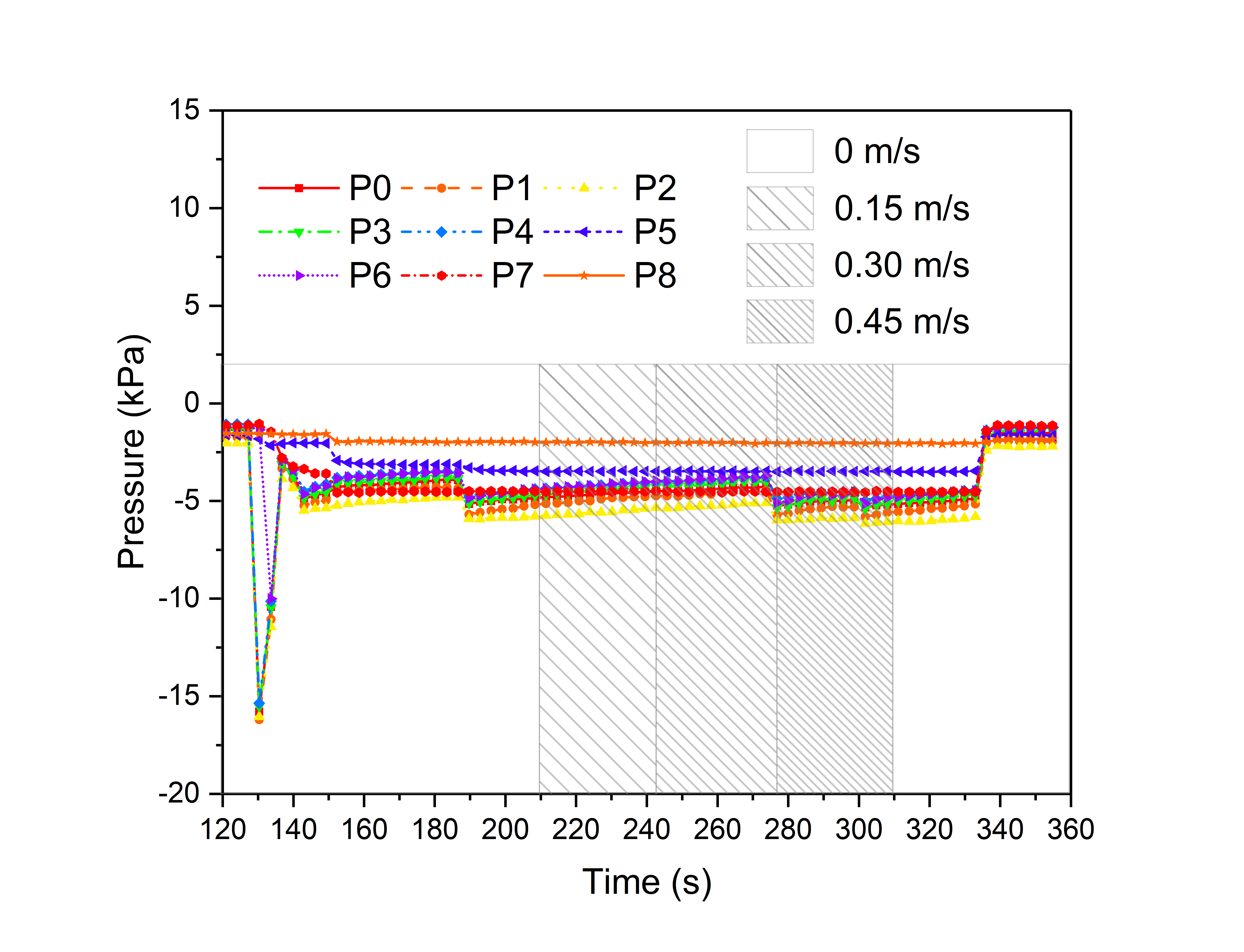


**S4-Fig 12.** **Suction dynamics of the adult male lamprey tagged as Blue 045 in Experiment 3.**


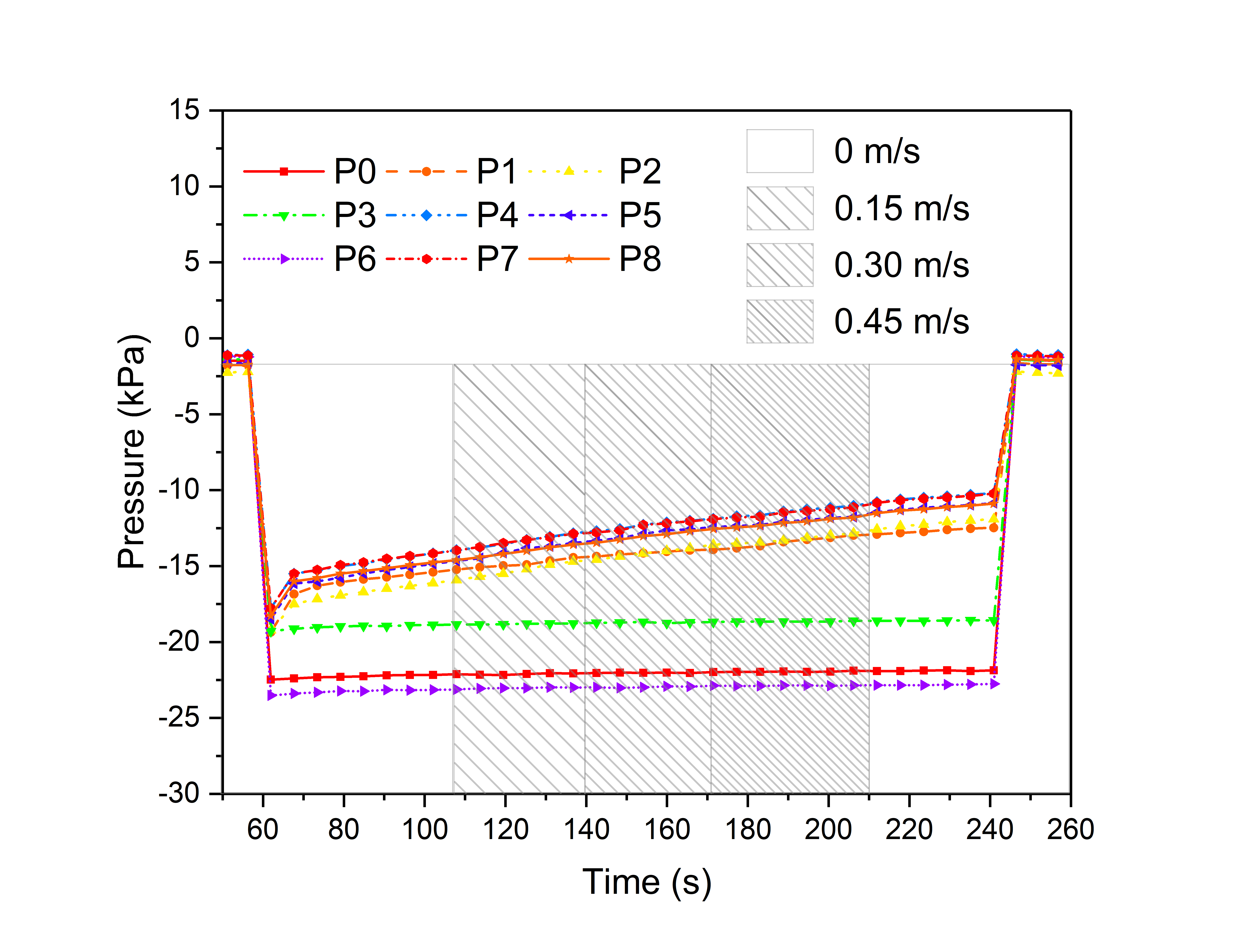


**S4-Fig 13.** **Suction dynamics of the adult male lamprey tagged as Pink 010 in Experiment 3.**


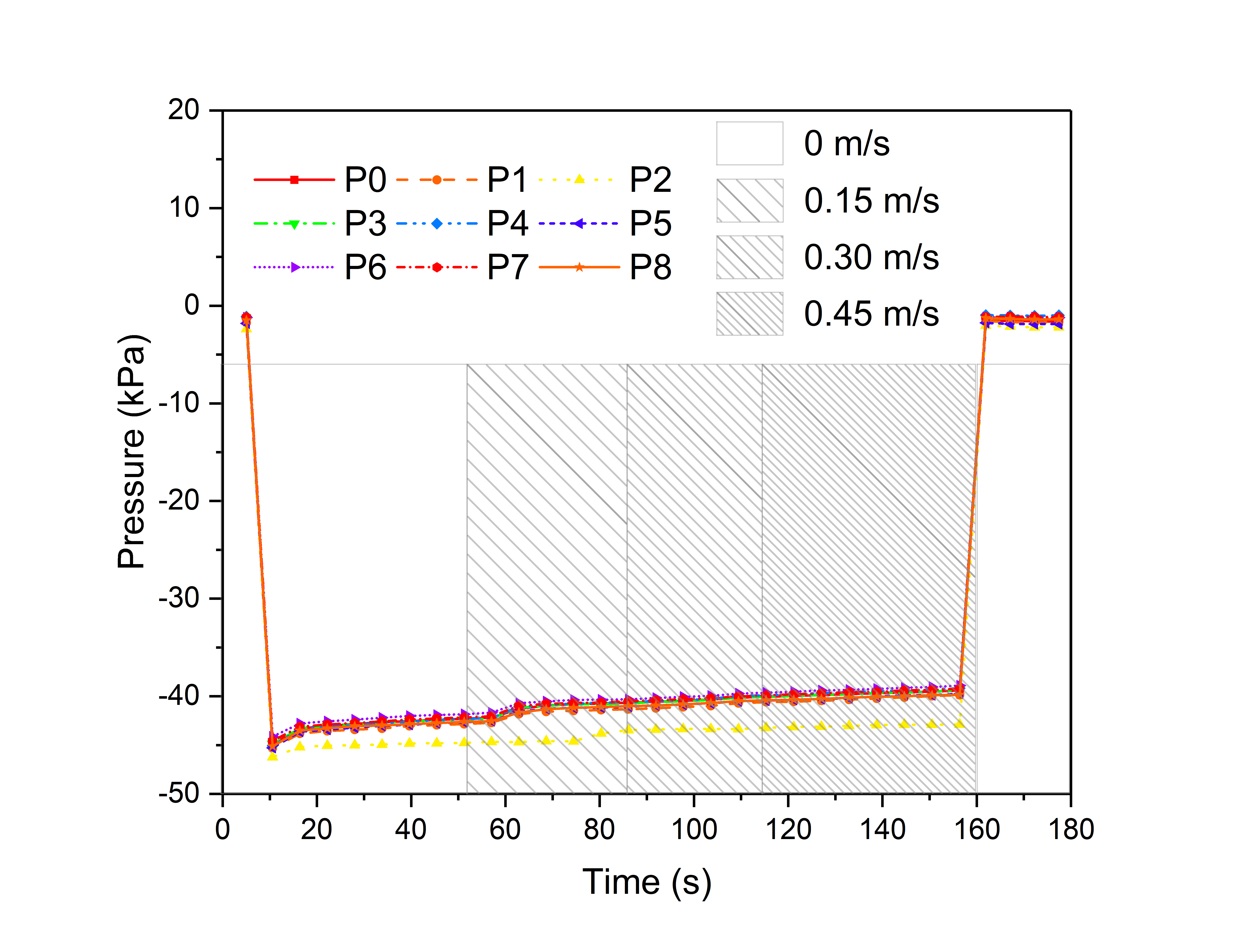


**S4-Fig 14.** **Suction dynamics of the adult male lamprey tagged as Pink 006 in Experiment 3.**


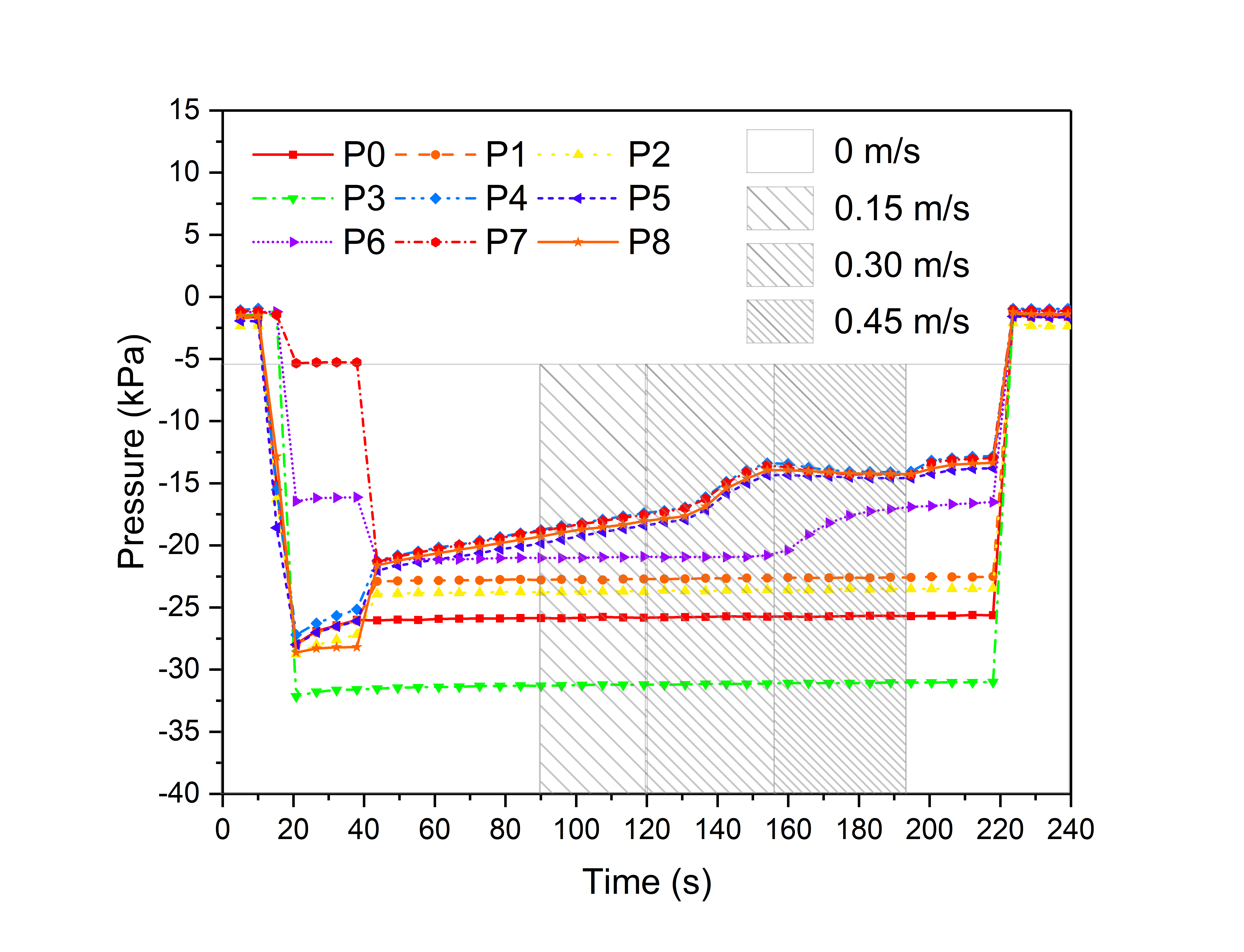


**S4-Fig 15.** **Suction dynamics of the adult male lamprey tagged as Pink 031 in Experiment 3.**


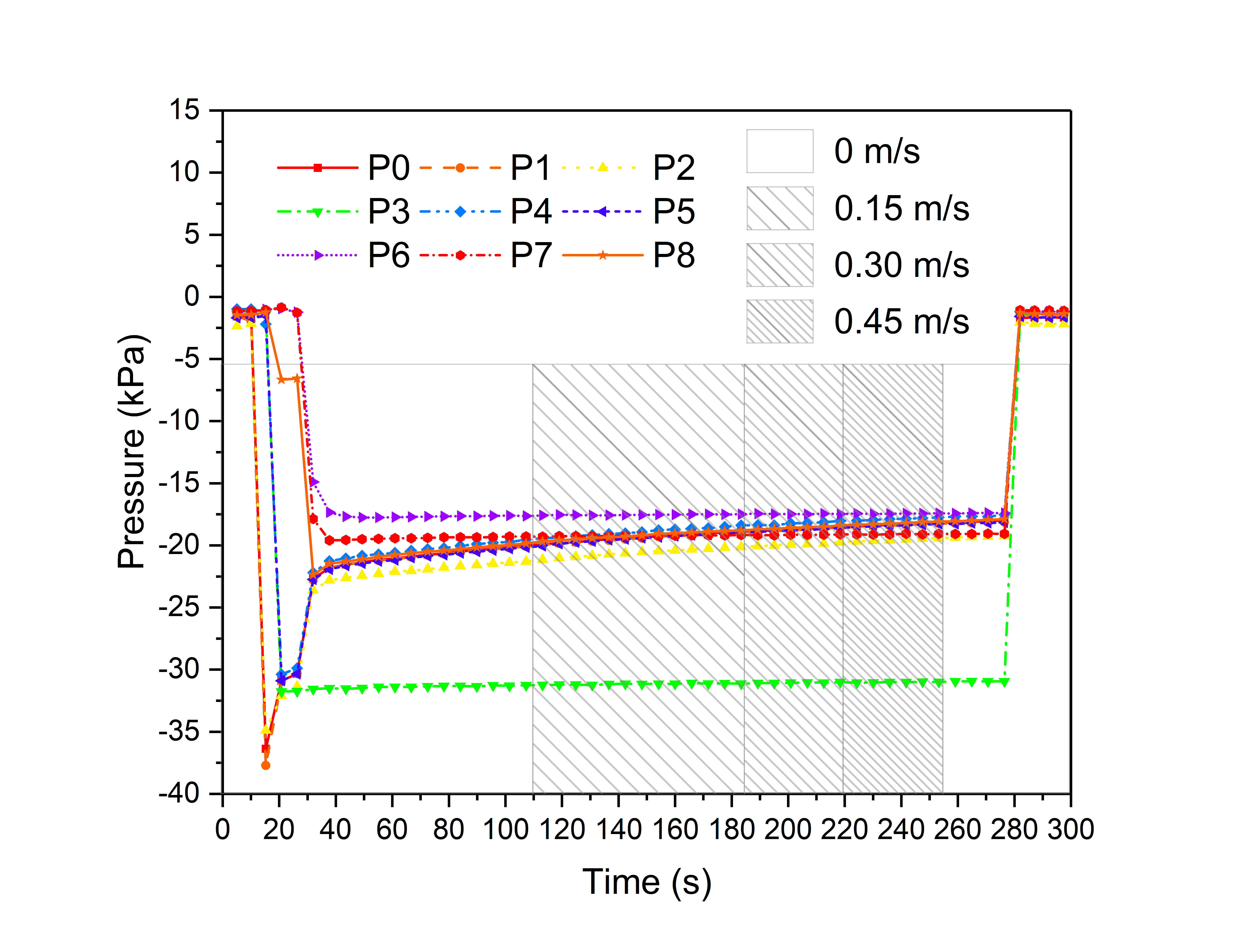


**S4-Fig 16.** **Suction dynamics of the adult male lamprey tagged as Blue 043 in Experiment 3.**
